# Community burden of undiagnosed HIV infection among adolescents in Zimbabwe following primary healthcare-based provider-initiated HIV testing and counselling: A cross-sectional survey

**DOI:** 10.1371/journal.pmed.1002360

**Published:** 2017-07-25

**Authors:** Victoria Simms, Ethel Dauya, Subathira Dakshina, Tsitsi Bandason, Grace McHugh, Shungu Munyati, Prosper Chonzi, Katharina Kranzer, Getrude Ncube, Collen Masimirembwa, Roslyn Thelingwani, Tsitsi Apollo, Richard Hayes, Helen A. Weiss, Rashida A. Ferrand

**Affiliations:** 1 MRC Tropical Epidemiology Group, London School of Hygiene and Tropical Medicine, London, United Kingdom; 2 Biomedical Research and Training Institute, Harare, Zimbabwe; 3 Harare City Health, Harare, Zimbabwe; 4 Department of Infectious Disease Epidemiology, London School of Hygiene and Tropical Medicine, London, United Kingdom; 5 Ministry of Health and Child Care, Harare, Zimbabwe; 6 African Institute of Biomedical Science & Technology, Harare, Zimbabwe; 7 Clinical Research Department, London School of Hygiene and Tropical Medicine, London, United Kingdom; Massachusetts General Hospital, UNITED STATES

## Abstract

**Background:**

Children living with HIV who are not diagnosed in infancy often remain undiagnosed until they present with advanced disease. Provider-initiated testing and counselling (PITC) in health facilities is recommended for high-HIV-prevalence settings, but it is unclear whether this approach is sufficient to achieve universal coverage of HIV testing. We aimed to investigate the change in community burden of undiagnosed HIV infection among older children and adolescents following implementation of PITC in Harare, Zimbabwe.

**Methods and findings:**

Over the course of 2 years (January 2013–January 2015), 7 primary health clinics (PHCs) in southwestern Harare implemented optimised, opt-out PITC for all attendees aged 6–15 years. In February 2015–December 2015, we conducted a representative cross-sectional survey of 8–17-year-olds living in the 7 communities served by the study PHCs, who would have had 2 years of exposure to PITC. Knowledge of HIV status was ascertained through a caregiver questionnaire, and anonymised HIV testing was carried out using oral mucosal transudate (OMT) tests. After 1 participant taking antiretroviral therapy was observed to have a false negative OMT result, from July 2015 urine samples were obtained from all participants providing OMTs and tested for antiretroviral drugs to confirm HIV status. Children who tested positive through PITC were identified from among survey participants using gender, birthdate, and location. Of 7,146 children in 4,251 eligible households, 5,486 (76.8%) children in 3,397 households agreed to participate in the survey, and 141 were HIV positive. HIV prevalence was 2.6% (95% CI 2.2%–3.1%), and over a third of participants with HIV were undiagnosed (37.7%; 95% CI 29.8%–46.2%). Similarly, among the subsample of 2,643 (48.2%) participants with a urine test result, 34.7% of those living with HIV were undiagnosed (95% CI 23.5%–47.9%). Based on extrapolation from the survey sample to the community, we estimated that PITC over 2 years identified between 18% and 42% of previously undiagnosed children in the community. The main limitation is that prevalence of undiagnosed HIV was defined using a combination of 3 measures (OMT, self-report, and urine test), none of which were perfect.

**Conclusions:**

Facility-based approaches are inadequate in achieving universal coverage of HIV testing among older children and adolescents. Alternative, community-based approaches are required to meet the Joint United Nations Programme on HIV/AIDS (UNAIDS) target of diagnosing 90% of those living with HIV by 2020 in this age group.

## Abstract 2

The community burden and stigma of HIV testing for adolescents are highlighted in a cross-sectional survey in Zimbabwe from Victoria Simms and colleagues.

## Author summary

**Why was this study done?:**

- In Zimbabwe, older children are rarely tested for HIV, and thus, they do not access the care they need.
- For 2 years in 7 clinics in Harare, every child aged 6–15 years who came into the clinic was offered an HIV test.
- The aim of this study was to find out how effective the clinic testing had been in finding undiagnosed children.

**What did the researchers do and find?:**

- The researchers did a house-to-house survey in Harare and tested children aged 8–17 years for HIV using an oral test.
- Of these 8–17-year-olds, 2.6% were HIV positive, and 38% of them had not been diagnosed.
- Children with HIV were less likely to have been diagnosed if they were 13 years of age or over, were in good health, and both their parents were alive.

**What do these findings mean?:**

- In order to diagnose all children with HIV, it is not enough to test every child who comes to a clinic, because some HIV-positive children either do not visit a clinic often or do not go with a guardian able to give consent for them to take an HIV test.
- Other methods, such as community-based testing or targeted tracing, must be tried to reach the children who cannot be tested in the clinic.

## Introduction

Worldwide, an estimated 1.8 million children under the age of 15 years are living with HIV, 90% of them in sub-Saharan Africa [1]. The incidence of HIV infection in infants has fallen substantially over the past decade because of the scale-up of interventions for prevention of mother-to-child HIV transmission (PMTCT), but coverage of PMTCT programmes remains suboptimal, with 150,000 infants infected worldwide in 2015 [2]. Importantly, an estimated 51% of children living with HIV were not receiving antiretroviral therapy (ART) in 2015, mainly because of low rates of HIV testing and, hence, underdiagnosis [1].

HIV testing for HIV-exposed infants is available as part of PMTCT programs, but only 51% of HIV-exposed infants access early infant diagnosis in high-HIV-burden countries [3], and diagnosis mainly occurs in healthcare facilities when children present with symptomatic disease in later childhood and adolescence [4–6]. Since 2007, the World Health Organization (WHO) has recommended provider-initiated HIV testing and counselling (PITC) in high-HIV-prevalence settings, whereby HIV testing is offered proactively to all clients attending a healthcare facility regardless of the reason for attendance [7]. PITC thus utilises the opportunity of a client presenting to a healthcare facility to offer HIV testing and counselling (HTC) and decrease the burden of undiagnosed HIV infection.

However, PITC relies on healthcare workers offering HIV testing. Healthcare workers often offer HTC to children selectively based on their perception of the client’s HIV risk and logistical constraints [8–10]. In addition, such an approach is limited to individuals attending health facilities and, for children, to those who attend with a guardian able to provide consent for the child to undergo HIV testing. Hence, PITC may not provide high enough coverage of HIV testing at the population level. This is particularly important for children, as facility-based PITC is often their only route to access HTC. Our hypothesis was that most undiagnosed children would present to a PHC over a 2-year period, and combined with optimised PITC to overcome facility-level barriers, this would result in a high proportion (at least 80%–90%) of HIV-positive children being identified. This study aimed to estimate at the community level the coverage of HIV testing provided by a PITC strategy with opt-out testing in primary healthcare clinics for older children and adolescents in Harare, Zimbabwe.

## Methods

Ethical approval for the study was obtained from the Medical Research Council of Zimbabwe (MRCZ/A/1676) and the Ethics Committees of Harare City Health Services, the Biomedical Research and Training Institute (BRTI) (AP 108/2012), and the London School of Hygiene and Tropical Medicine (6305).

Between January 2013 and January 2015, children aged 6 to 15 years attending primary healthcare clinics (PHCs) for any reason in 7 communities in southwest Harare were offered PITC. Every child who attended with a guardian, was not in HIV care, and had not had a documented HIV test within the last 6 months was offered HIV testing by clinic staff, supported by the study team. A guardian was defined as the main caregiver, as determined by the clinic staff. National HIV testing guidelines were followed, and the test results were available within 20 minutes [11]. Children with a positive test who lived locally were offered HIV care and invited to enrol into a cohort study. Following initial low levels of testing, an opt-out HTC approach was adopted in July 2013, and supplemental staff and test kits were provided to improve testing rates at the study clinics [9]. The uptake, prevalence, and yield of HIV testing at clinics during the PITC programme have been reported previously [11]. Briefly, of 10,673 clinic attendees aged 6–15 years over the 2-year period who were not in HIV care or recently tested and who attended with a guardian able to provide consent to test, 8,468 (79.3%) underwent HTC, and among these, the HIV prevalence was 5.1% [9,11].

Following the 2 years of optimised PITC, a community-based survey was conducted in the 7 communities to estimate the prevalence of undiagnosed HIV among children aged 8 to 17 years and investigate the impact of PITC. This age group was selected to ensure exposure over the whole intervention period.

### Sampling

The sampling frame was a list of census enumeration areas (CEAs) in the study communities defined as the smallest delimited census sampling area used for the 2012 National Census. CEAs tagged with the census geocode for private housing were selected from the 7 communities using simple random sampling. All households in the selected CEAs were enumerated. Any household with at least 1 member aged 8 to 17 years was defined as eligible. Children were not eligible if they were not staying in the household or not available at the time of enumeration and for the following 2 weeks. All eligible children were invited to participate. Written informed consent from the guardian was obtained for all participants, except for emancipated minors (defined as those who were married, were living with sexual partners, had children, or were heads of households) who were able to consent independently. Age-appropriate, written assent was obtained from survey participants prior to recruitment. Two different assent forms (1 for 8–12-year-olds and 1 for those aged 13–17 years) with information about the survey appropriate to the level of understanding were used to obtain assent. Verbal assent with an independent witness was obtained if the participant was not able to sign the assent form.

### Data collection

An appointment was made to visit the eligible child at home. Up to 2 repeat visits were made if the child was not available. An interviewer-administered questionnaire with electronic data entry was used to record sociodemographic details and history of HIV testing and treatment. The location of the home was recorded using latitude and longitude. Patient-held records and documentation of previous HIV test results and treatment were used to confirm the information provided. All participants underwent anonymised HIV testing with an oral mucosal transudate (OMT) test (Oraquick, Orasure Technologies, Pennsylvania, United States). Those with no documentary evidence of an HIV test were given written information about HIV testing and were given a voucher to access diagnostic HIV testing at a local PHC using standard clinic procedures. The OMT result window was covered with a sticker that was only removed in the trial office. The HIV test results were delinked from personal identifiers, and therefore, an individual’s test results could not be identified by the study team or be made available to participants.

In June 2015, a participant with documented evidence of taking ART was noted to have a false-negative HIV test result, probably due to viral suppression. The study team made the decision to collect urine samples for measurement of antiretroviral (ARV) levels from all participants in the remaining CEAs, which was enacted from 9 July 2015. Participants were provided with a disposable plastic container in which to void urine. A minimum of 10 ml of urine was decanted into a prelabelled 50-ml Falcon tube. Samples were transferred within 6–8 hours to the laboratory, where they were stored at −20°C until processing. Reverse-phase high-performance liquid chromatography interfaced with mass spectrometry was validated to detect lamivudine, tenofovir, nevirapine, and ritonavir. All ART regimens in Zimbabwe following national guidelines included at least 1 of these drugs, and in this setting, the probability of a child receiving HIV care from private providers was low [12]. Samples were pooled in batches of 20, with individual testing of each sample if a pool tested positive for any ART drug.

### Data analysis

Data were collected on Nexus 7 electronic tablets using Open Data Kit and transferred to an MS Access database. HIV test results were recorded on paper forms and entered into an MS Access database using Cardiff TELEFORM Intelligent Character Optical Mark Recognition Software (version 10.9). All analyses were performed using Stata version 14.1 (StatCorp, Texas, US). Survey methods and outcomes are detailed in the protocol for the Zimbabwe Study for Enhancing Testing and Improving Treatment of HIV in Children (ZENITH), of which this survey is 1 part. The statistical analysis plan was written after data collection was completed. Analyses were weighted by community using inverse probability weighting to allow for CEAs that were not surveyed because of lack of time. The prevalence of undiagnosed HIV was estimated in the whole sample and also in the subset with a urine test result. In both cases, participants were defined as HIV positive if they (1) had a positive OMT result, (2) self-reported they were HIV positive, or (3) had ART detected in their urine. HIV-infected participants were considered undiagnosed if they were OMT negative and did not have either a positive urine test or documented evidence of a previous positive HIV test result.

The primary outcome was the proportion of all participants living with HIV who were undiagnosed. The secondary outcome was the proportion of participants living with HIV who were undiagnosed among the subsample who also had urine ARV test results. Based on 2012 census data, and assuming an HIV prevalence of 3% among 8–17-year-olds, an intracluster correlation of 0.3, and a 20% refusal to participate, we predicted a sample of 150 CEAs (6,562 children) would provide 10% precision around an estimated 40% prevalence of undiagnosed HIV at a 5% significance level.

The prevalence of participants with HIV and among these the proportion with undiagnosed HIV were estimated with 95% confidence intervals (95% CI) allowing for clustering by community with CEA as a primary sampling unit, with inverse probability weighting to allow for CEAs that were not surveyed. Characteristics of participants and households were described. Logistic regression was used to identify factors associated with undiagnosed HIV among participants living with HIV and factors associated with HIV among all participants, with adjustment for multistage sampling and inverse probability weighting. Factors associated with outcomes at 10% significance in univariate analysis were taken forward into multivariate analysis using mixed-effects logistic regression adjusting for community and CEA as random effects, with age and gender selected a priori. Participants diagnosed during PITC were linked to the survey data using probabilistic record linkage on birthdate, gender, and location of home. The number of children living with HIV diagnosed with PITC was compared with an extrapolation of the number living with HIV in the community to estimate the proportion reached through PITC.

## Results

One CEA was replaced because it was an undeveloped plot of land, and 6 were replaced because the Zimbabwe Central Statistics Office could not provide maps. Of the 150 selected CEAs, 130 (86.7%) were surveyed (S1 Table). Eighteen CEAs in 1 area were not surveyed within the time limit of the survey, and 2 contained police private accommodation, which the study team was not given permission to survey. The 18 missing CEAs were not known to be different from those included in the survey. Urine samples were collected in 71 (54.6%) CEAs, and analyses of this subset were weighted using inverse probability weighting to represent the whole population within the 6 communities that collected urine samples. The median number of households per CEA was 23.5 (IQR 18–29). The survey team visited 8,300 households, of which 668 were vacant, 138 refused enumeration, and 7,494 (90.3%) were enumerated, with 4,251 containing at least 1 child aged 8–17 years. Refusing households were evenly distributed by community and over time. In terms of demographic characteristics, there was no difference between children whose caregivers refused consent, or who did not assent, to the trial and those who participated.

### Participant characteristics

**Fig 1:**
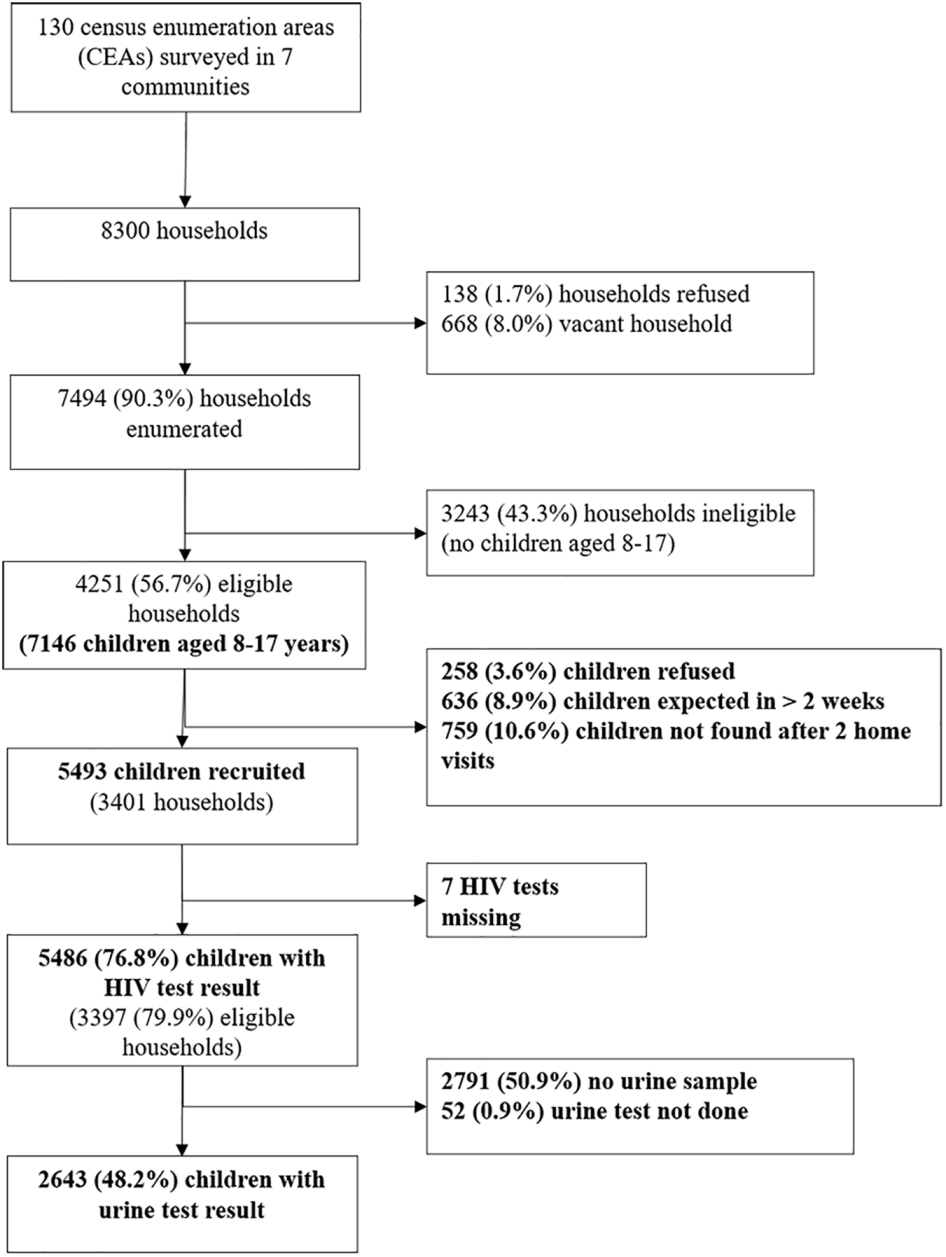
Flow of participant enrolment.

Of 7,146 children in 4,251 eligible households, the survey recruited 5,486 children from 3,397 households between 23 February 2015 and 17 December 2015 (Fig 1). The median size of participating households was 5 (IQR 4–6), consisting of adults (median 2, IQR 2–3), children aged under 8 years (median 1, IQR 0–1), and children aged 8–17 years (median 2, IQR 1–2). The sociodemographic characteristics of households are shown in Table 1. About half of the participants (53.2%) were female, and about a quarter (26.9%) had a main carer who was not a biological parent. Urine samples were requested from everyone enrolled from 9 July 2015 onwards, and 2,695 (82.9%) of 3,249 participants approached (49.1% of all participants) provided a urine sample. Participants who refused to give a urine sample were more likely to be older and female than those who agreed (53.1% of refusers aged 13–17 years compared to 47.0% of donors [*p* = 0.009]; 58.7% female versus 52.5% [*p* = 0.008]). There was no association of refusal to provide urine with self-reported HIV prevalence.

**Table 1:** Baseline characteristics of surveyed households and participants.

Variable		*N* (%) of participants	*N* (%) of households
		*N* = 5,486	*N* = 3,397
**Household-level characteristics**			
**Number of children aged 8–17 years in household**	1	1,681 (30.6)	1,680 (49.5)
	2	2,046 (37.3)	1,142 (33.6)
	3	1,162 (7.0)	429 (12.6)
	4–9	597 (10.9)	146 (4.3)
**Has children aged <8 years**	Yes	3,227 (58.8)	2,031 (59.8)
**Child-headed household (<18 years)**		13 (0.2)	8 (0.2)
**Age of household head**	Years (median [IQR])	42 (36–51)	41 (35–50)
	<25	113 (2.1)	84 (2.5)
	25–39	1,988 (36.2)	1,316 (38.8)
	40–59	2,648 (48.3)	1,582 (46.6)
	60+	736 (13.4)	414 (12.2)
**Education level of household head**	None	85 (1.6)	46 (1.4)
	Primary	387 (7.1)	226 (6.7)
	Secondary	4,278 (78.0)	2,674 (78.7)
	University	423 (7.7)	256 (7.5)
	Training college	312 (5.7)	194 (5.7)
**Household ownership of dwelling**	Own the dwelling	2,785 (50.8)	1,618 (47.6)
	Rent whole of dwelling	825 (15.0)	530 (15.6)
	Rent part of dwelling (i.e., lodger)	1,577 (28.8)	1,059 (31.2)
	Use dwelling without paying rent	298 (5.4)	189 (5.6)
**Number of people earning an income**	0	2,316 (42.2)	1,419 (41.8)
	1	2,664 (48.6)	1,680 (49.5)
	2–4	505 (9.2)	297 (8.7)
**Monthly income (USD)**	No regular income	2,752 (51.5)	1,696 (51.2)
	<USD 100	23 (0.4)	17 (0.5)
	USD 100–200	200 (3.7)	127 (3.8)
	USD 201–500	1,472 (27.5)	965 (29.1)
	USD 501–900	780 (14.6)	441 (13.3)
	>USD 900	118 (2.2)	68 (2.1)
**Owns a fridge**	Yes	4,760 (87.8)	2,896 (85.3)
**Owns a car**	Yes	903 (16.5)	530 (15.6)
**Owns a TV**	Yes	5,308 (96.8)	3,267 (96.2)
**Owns a mobile phone**	Yes	5,443 (99.2)	3,366 (99.1)
**Nontrauma death in household in past year**	Yes	83 (1.5)	52 (1.5)
**Participant-level characteristics**			
**Gender**	Female	2,921 (53.2%)	
**Age**	Years (median [IQR])	12 (10–15)	
**Education**	In low grade for age/never been to school	1,811 (33.0)	
**Marital status**	Ever married or lived together as if married	55 (1.00)	
**Orphanhood**	Both parents alive	4,567 (83.3)	
	Single orphan	719 (13.1)	
	Double orphan	200 (3.7)	
**Caregiver**	Parent	4,010 (73.1)	
	Aunt/uncle	538 (9.8)	
	Grandparent	595 (10.9)	
	Sibling	212 (3.9)	
	Other relative	89 (1.6)	
	Nonrelative	42 (0.8)	
**Residence in current household**	Years (median [IQR])	10.0 (8–14)	
**TB**	Ever treated	62 (1.1)	
**Hospital admission**	Ever admitted	332 (6.1)	
**Self-report health**	Excellent/good	5,269 (96.1)	
	Fair/poor	217 (3.9)	
**Previously tested for HIV**	Yes	633 (11.6)	

TB, tuberculosis; USD, US dollar.

### Previous HIV tests

Out of 633 children who reported previously testing for HIV, 284 (44.9%) tested in a primary care clinic, usually (*n* = 231) the PITC clinic closest to their home. Additionally, 152 (24.0%) reported testing in a hospital, and 76 (12.0%) in a mobile testing centre or other community outreach. Less than half (*n* = 277, 44.2%) reported that they tested during the PITC intervention period of 2013–2014. Sixty-five (10.3%) reported that they tested positive, and 62/65 (95.4%) produced proof of their HIV status. Three children had missing data for ART, and 60/62 reported they were taking ART.

### Prevalence of HIV and undiagnosed HIV infection

Of 5,486 children with OMT results, 122 (2.2%) had a positive result. Of these 122 participants, 53 (43.4%) were undiagnosed (i.e., no evidence of ARVs in urine and no positive self-report).

A total of 44 participants had a positive urine ARV result. Of these, OMT identified HIV in 36 participants, giving a sensitivity of 81.8% for HIV detection in patients taking ART against the urine result (95% CI 67.3%–91.8%). The families of 23 participants (52.3%, 95% CI 36.7%–67.5%) claimed that the children were HIV negative or had not been tested (i.e., known HIV infection that was not reported in the survey). In addition to the 122 children with a positive OMT result, 8 participants had a negative OMT result but a positive urine ART result, and a further 11 had a negative OMT result but self-reported themselves as HIV positive, although their urine test was either negative (*n* = 2) or not done (*n* = 9) (Table 2). Thus, the total number of children with HIV (based on an OMT result, self-report, or ART detected in urine) was 141, and the HIV prevalence was 2.60% (95% CI 2.17%–3.11%) (Table 3). The prevalence of undiagnosed HIV infection among all children with HIV was 37.7% (95% CI 29.8%–46.2%). The corresponding results among the subsample with urine test results were similar (HIV prevalence 2.80%, 95% CI 2.02%–3.87%; and undiagnosed HIV prevalence 34.7%, 95% CI 23.5%–47.9%). Compared to children aged 8–12 years, adolescents aged 13–17 years had higher HIV prevalence and were more likely to be undiagnosed, although confidence intervals overlapped. Unweighted results were very similar to the weighted results (S3 Table).

**Table 2:** Diagnosed HIV as defined by OMT result, self-report, and urine ART result.

		ART detected in urine	ART not detected	Urine test not done	Total
**OMT positive**	**Self-report as HIV positive**	18	5	28	**51**
	**Self-report as HIV negative**	2	3	8	**13**
	**Self-report as never tested**	16	22	20	**58**
	**Total**	**36**	**30**	**56**	**122**
**OMT negative**	**Self-report as HIV positive**	3	2	9	**14**
	**Self-report as HIV negative**	0	191	364	**555**
	**Self-report as never tested**	5	2,376	2,414	**4,795**
	**Total**	**8**	**2,569**	**2,787**	**5,364**
**Total**		**44**	**2,599**	**2,843**	**5,486**

Yellow indicates participants defined as living with diagnosed HIV, either by ART detected in urine, self-report, or OMT. Red indicates participants defined as living with undiagnosed HIV.

ART, antiretroviral therapy; OMT, oral mucosal transudate.

**Table 3:** HIV prevalence and proportion of undiagnosed HIV infection.

Participants	Age group (years)	*N*	HIV positive	Weighted HIV prevalence (95% CI)	Undiagnosed HIV	Weighted prevalence of undiagnosed HIV (95% CI)
**All participants**	**All**	**5,486**	**141**	**2.60% (2.17%–3.11%)**	**53**	**37.7% (29.8%–46.2%)**
	8–12	2,835	60	2.15% (1.61%–2.87%)	17	29.2% (17.5%–44.6%)
	13–17	2,651	81	3.07% (2.43%–3.87%)	36	43.9% (32.6%–55.9%)
**Only participants with a urine test result**	**All**	**2,643**	**76**	**2.80% (2.02%**–**3.87%)**	**25**	**34.7% (23.5%–47.9%)**
	8–12	1,401	36	2.53% (1.71%–3.70%)	10	28.1% (15.1%–46.2%)*
	13–17	1,242	40	3.10% (2.13%–4.51%)	15	40.7% (24.1%–59.7%)

*Two strata were combined, as 1 community had only 1 HIV-positive participant aged 8–12 years with a urine test result.

### Risk factors for HIV and undiagnosed HIV infection

Among the 141 participants with HIV, those who were older, had both parents still living, reported good health, had never been admitted to hospital or treated for TB, did not have recurring skin problems, or had a higher household income were more likely to be undiagnosed (Table 4). In a multivariate model, death of a parent (OR = 0.39, 95% CI 0.18–0.88) and poor health (OR = 0.10, 95% CI 0.02–0.48) were associated with reduced odds of undiagnosed HIV, and older age (OR = 0.25, 95% CI 1.07–5.89) was associated with increased odds. Tuberculosis (TB) history was removed from the model because of collinearity with hospital admission. HIV positivity was associated with being an orphan, having a nonparent caregiver, being in a lower grade at school for age, having an older head of household, hospital admission, and being aged 13 years and above in univariate analysis (S2 Table). In multivariate analysis, independent risk factors for HIV were death of a parent, hospital admission, and being in a lower school grade for age. In households in which a child was living with HIV, the household head was more likely to report being comfortable sharing food with an HIV-positive child.

**Table 4:** Factors associated with undiagnosed HIV among participants living with HIV (*N* = 141).

Risk factor	Categories	Diagnosed	Undiagnosed	Univariate		Multivariate	
		*n* = 88	*n* = 53	OR (95% CI)	*p*	OR (95% CI)	*p*
**Gender**	Male	46 (52.3)	24 (45.3)	1	–	1	–
	Female	42 (47.7)	29 (54.7)	1.32 (0.67–2.62)	0.42	1.00 (0.45–2.22)	0.99
**Age group (years)**	8–12	43 (48.9)	17 (32.1)	1	–	1	–
	13–17	45 (51.1)	36 (67.9)	2.02 (0.99–4.13)	0.05	2.52 (1.07–5.89)	0.03
**Parent died**	No	43 (48.9)	34 (64.2)	1	–	1	–
	Yes	45 (51.1)	19 (35.9)	0.53 (0.27–1.08)	0.08	0.39 (0.18–0.88)	0.02
**Marital status**	Never married/lived together	87 (98.9)	51 (96.2)	1	–		
	Ever married/lived together	1 (1.1)	2 (3.8)	3.41 (0.30–38.60)	0.32		
**Education**	Normal/high grade for age	39 (44.3)	25 (47.2)	1	–		
	Low grade for age/never been to school	49 (55.7)	28 (52.8)	0.89 (0.45–1.77)	0.74		
**Caregiver**	Biological parent	52 (59.1)	29 (54.7)	1	–		
	Not a parent	36 (40.9)	24 (45.3)	1.20 (0.60–2.38)	0.61		
**Share food with HIV+ child**	Comfortable	88 (100.0)	48 (90.6)				
	Uncomfortable	0	5 (9.4)				
**Age of HH head (years)**	20–24 (reference group)	2 (2.3)	3 (5.7)	0.68 (0.44–1.07)*	0.10		
	25–39	20 (23.9)	18 (34.0)				
	40–59	45 (51.1)	23 (43.4)				
	60+	20 (22.7)	9 (17.0)				
**Education of HH head**	Secondary/further	74 (84.1)	53 (100.0)				
	None/primary	14 (15.9)	0				
**HH monthly income**	No regular salary (reference group)	57 (64.8)	29 (54.7)	1.30 (0.97–1.75)*	0.08	1.22 (0.88–1.68)*	0.24
	USD 1–200	3 (3.4)	2 (3.8)				
	USD 201–500	22 (25.0)	10 (18.9)				
	>USD 500	6 (6.8)	12 (22.6)				
**Hospital admission**	Never admitted	59 (67.1)	45 (84.9)	1	–	1	–
	Ever admitted	29 (33.0)	8 (15.1)	0.36 (0.15–0.87)	0.02	0.52 (0.20–1.38)	0.19
**Recurring skin problems**	No	69 (78.4)	49 (92.5)	1	–	1	–
	Yes	19 (21.6)	4 (7.6)	0.29 (0.09–0.94)	0.04	0.42 (0.12–1.53)	0.19
**TB treatment history**	Never treated for TB	70 (79.6)	51 (96.2)	1	–		
	Ever treated	18 (20.5)	2 (3.8)	0.15 (0.03–0.67)	0.01		
**Overall health**	Excellent/good	62 (70.5)	51 (96.2)	1	–	1	–
	Fair/poor	26 (29.6)	2 (3.8)	0.09 (0.02–0.41)	<0.01	0.10 (0.02–0.48)	<0.01

* Odds ratio for trend.

### Impact of PITC on the community burden of undiagnosed HIV

We extrapolated from our sample to the target population using the CEA sampling fraction (7.57%) and the proportion of eligible households (90.2%, 95% CI 89.6%–90.9%) and children (76.8%, 95% CI 75.8%–77.8%) surveyed. We estimate there were around 104,533 children aged 8–17 years residing in the 7 communities, of whom 2,717 (2.60%) were living with HIV and 1,024 (37.7%) were undiagnosed. The error around the estimate of 1,024, using 95% confidence intervals for all 5 variables, is 562–1,825. PITC at the PHCs over 2 years identified 449 children [11], but of these, 9 later were found to be already diagnosed and on ART, 2 were aged over 15 years, and 24 did not live in the study communities, leaving 414. Extrapolating from the survey and disregarding migration, we estimate that before PITC there were therefore 1,438 (1,024 plus 414) children (range 976–2,239) living with undiagnosed HIV. Using these estimates, PITC over 2 years identified approximately 414/1,438 or 29% (18%–42%) of previously undiagnosed children.

All children diagnosed through PITC who lived locally and for whom consent was given were followed up in a cohort study (*n* = 385). Of these, 362 were still living in the community in 2015, and we calculated that we could expect 19 (95% CI 11–28) of them to be included in the survey if they were randomly distributed. Using probabilistic record linkage on date of birth, gender, and home location of children who were diagnosed through PITC and were in clinic, we identified 13 close matches and 5 likely matches.

## Discussion

This study demonstrates a substantial burden of undiagnosed HIV infection among older children and especially among adolescents. More than a third of children aged 8–17 years living with HIV in the community remained undiagnosed after 2 years of PITC.

The uptake and prevalence of HIV testing through PITC have been reported previously and are repeated here to aid interpretation of the survey results. Of 10,673 children aged 6–15 years who did not know their HIV status and attended the study PHCs with a consent-capable guardian over 2 years, 20.7% did not undergo HIV testing [9]. However, a further 2,871 children attended the clinic alone or were accompanied by a caregiver deemed unsuitable by healthcare workers to give consent for the child to undergo HIV testing and therefore were not eligible for HIV testing [9]. Thus, even with optimised PITC, overall only 62.5% of attendees with unknown HIV status were tested, which may explain why children with HIV remained undiagnosed. Even if PITC is available at facilities, the success of this approach depends on clients attending a health facility—and in the case of minors, attending with guardians who can provide consent. In this survey, 43% of children living with HIV had a primary caregiver who was not a biological parent. Such caregivers, usually extended family members, may not be able to give consent for the child to be tested. Additionally, children may change guardian frequently or may attend clinic alone.

Children with HIV who survive infancy undiagnosed often develop minor infections such as upper respiratory tract and skin infections before developing advanced disease [6,13]. These minor infections may prompt presentation to a primary healthcare facility but are not severe enough to require admission to hospital. Our hypothesis was that most undiagnosed children would present to a PHC over a 2-year period, and combined with optimised PITC to overcome facility-level barriers, this would result in identification of a high proportion (at least 80%–90%) of children living with HIV. Instead, extrapolation from the survey suggests that only two-thirds of children living with HIV were identified. It is likely that children more prone to infections had been diagnosed prior to the study, at primary or secondary care clinics. Children with undiagnosed HIV infection may be those who have few obvious risk factors such as orphanhood and fewer symptoms, making them less likely to present at health care facilities. Among participants with HIV in this study, orphanhood and poor overall health were strongly associated with lower odds of being undiagnosed. The findings are unlikely to be due to recent migration into the area, as 91.2% of participants had been living in the area for at least 2 years. Some HIV cases in this adolescent population may have been due to more recent behavioural infection rather than perinatal transmission, and so they would not have been attending clinics or perhaps infected during PITC. However, this is unlikely to have been a major cause of HIV, because there was no difference in prevalence between girls and boys in either younger or older age groups.

Over half of the children living with HIV were in a low school grade for their age or had never been to school, compared to a third of HIV-negative peers. Being in a low school grade for age was associated with living with HIV and was no different among those who were diagnosed and undiagnosed.

Between 13 and 18 children diagnosed through PITC were identified in the survey, which is close to the number that would be expected. However, only 3 children in the survey reported being diagnosed through PITC. The other cohort-matched children either did not reveal their HIV status (but were diagnosed by ART in their urine) or said they were diagnosed before the PITC period. This indicates that self-reporting of this information is not accurate, as families both under-report children being HIV positive and claim that they were diagnosed longer ago than they actually were. Some children on ART who did not have a urine test may have tested negative using the OMT, so low self-reporting is likely to have caused an underestimate of HIV prevalence and of the proportion of HIV to be undiagnosed. A possible reason for nonreport of children’s HIV diagnosis is that the caregiver did not want to disclose the child’s status to other family members.

To our knowledge, this is the first study to provide estimates of undiagnosed HIV among adolescents at the community level. The strengths of the study were a large sample size, a representative sample, high participation rates, and the use of a sensitive urine test for ARVs to validate the OMT tests. During the 2 years of the intervention, there were no changes to HIV policy or practice that are likely to have affected the outcome. We acknowledge several limitations. A baseline survey was not done, as this would have constituted a mass testing intervention and affected the prevalence of undiagnosed HIV in the population, so our estimates of how the prevalence of undiagnosed HIV might have changed over time are based on backward projections. Undiagnosed HIV infection was defined using 3 different outcome measures, each of which had shortcomings. Under-reporting of known HIV infection is an inherent problem when using self-report. Patient-held clinical records were used to confirm 62/65 previous diagnoses, so false-positive reports are unlikely. False-negative self-reports of HIV status did occur; most children who were matched to PITC positive tests did not report they were HIV positive, and among the 13 participants with a positive OMT result who reported they were HIV negative, 2 had traces of ART in their urine. OMT testing has been shown to have high sensitivity (99.4%) among untreated individuals [14,15] and was selected as the preferable test because of its noninvasiveness and thus expected higher acceptability among children, as shown in the pilot. However, during the survey we discovered that the sensitivity of the OMT test was lower among those who were on ART. Urine ART tests were used to address the issue of false-negative OMT test results in participants on long-term ART but were only done in half of the sample and could only identify those who were on treatment. The estimate of the proportion of children diagnosed through PITC depends on extrapolation from the survey sample to the entire population and has wide confidence intervals. Participation was high, but those who refused to participate may have been less likely to attend PHCs or accept HIV testing, which would have led to an underestimate of undiagnosed HIV prevalence. Finally, the survey was conducted in an urban, high-density setting, well served by PHCs. The proportion of undiagnosed HIV may be higher in rural settings, where access to clinics may be more limited.

Five participants self-reported they were HIV positive and on treatment but had either low (below threshold) or no ART in their urine. They may have been partially adherent, nonadherent, or not started treatment. The fact that all 5 were aged 14–17 years and diagnosed at least 4 years previously suggests that partial adherence or nonadherence is more likely. With OMT test specificity less than 100% and low prevalence of HIV infection in this age group, a proportion of positive results will be false. A meta-analysis in 2012 found OMT test specificity was 99.86% [14], which in a population with 2.6% HIV prevalence gives a positive predictive value of 95.0%, meaning we can expect that approximately 6/122 positive OMT test results were false positives. This is counterbalanced by the fact that OMT test sensitivity is lower in children taking ART. Confirmatory testing of positive samples could have addressed the issue of false positives, but the pilot study found that fingerprick blood tests were less acceptable than noninvasive OMT tests.

According to our extrapolation, 2 years of optimised PITC in primary healthcare facilities in a high-HIV-prevalence setting reduced the proportion of undiagnosed HIV in children and adolescents living with HIV to 38%. However, more than one-third of HIV-infected children and adolescents at the population level remained undiagnosed. Community-based testing and counselling approaches are needed to reach the children and adolescents who either do not make contact with health facilities or attend without a guardian.

Community-based approaches might include school-based testing, testing at youth activities, or programmes targeted towards those children most likely to be undiagnosed. As we previously showed in PHCs [16], targeted testing might be more efficient and effective than the blanket household testing employed in this survey. In addition, engagement with ministries of health to lower the age of consent for HIV testing would increase the number of adolescents able to test.

## Supporting information

S1 STROBE Checklist Strengthening the Reporting of Observational Studies in Epidemiology (STROBE) checklist. (DOCX)

S1 Table Sampling strategy for census enumeration areas (CEAs). (DOCX)

S2 Table Factors associated with HIV among all participants (*N* = 5,486). (DOCX)

S3 Table Unweighted HIV prevalence and proportion with undiagnosed HIV. (DOCX)

S1 Text Biomedical Research and Training Institute–Zimbabwe Study for Enhancing Testing and Improving Treatment of HIV in Children (BRTI-ZENITH) Household Baseline Form ZP01. (PDF)

S2 Text Individual questionnaire: Prevalence survey ZP02. (PDF)

S3 Text BRTI-ZENITH Anonymous HIV Test Form ZP03. (PDF)

S4 Text BRTI-ZENITH Diagnostic HIV Test ZP04. (PDF)

S5 Text ZENITH Protocol v4.0, 1 June 2015. (DOC)

S6 Text Statistical analysis plan v1.2, 7 April 2016. (DOCX)

## Abbreviations

ART: antiretroviral therapy
ARV: antiretroviral
BRTI: Biomedical Research and Training Institute
CEA: census enumeration area
HTC: HIV testing and counselling
OMT: oral mucosal transudate
PHC: primary health clinic
PITC: provider-initiated testing and counselling
PMTCT: prevention of mother-to-child HIV transmission
TB: tuberculosis
STROBE: Strengthening the Reporting of Observational Studies in Epidemiology
UNAIDS: Joint United Nations Programme on HIV/AIDS
USD: US dollar
WHO: World Health Organization
ZENITH: Zimbabwe Study for Enhancing Testing and Improving Treatment of HIV in Children
