# Supplementary material for: Community burden of undiagnosed HIV infection among adolescents in Zimbabwe following primary healthcare-based provider-initiated HIV testing and counselling: A cross-sectional survey
Source: PLoS Med. 2017 Jul 25;14(7):e1002360. doi: 10.1371/journal.pmed.1002360 (PMC5526522; doi:10.1371/journal.pmed.1002360)
Supplement: S1 Table — (DOCX) [file pmed.1002360.s002.docx]

## Supplementary Table 1: Sampling strategy for census enumeration areas (CEAs)

| **Community** | **Budiriro** | **Dzivarasekwa** | **Glen Norah** | **Glenview** | **Highfield** | **Kuwadzana** | **Mufakose** | **Total** |
| --- | --- | --- | --- | --- | --- | --- | --- | --- |
| **Total number of CEAs** | 371 | 152 | 180 | 298 | 250 | 338 | 128 | 1717 |
| **Number of CEAs selected for survey** | 27 | 12 | 15 | 22 | 23 | 40 | 11 | 150 |
| **Changes** | 13 not done - ran out of time |  | 5 not done – ran out of time |  | 2 not done – high security |  |  | 20 not done |
| **Number of CEAs in survey** | **14** | **12** | **10** | **22** | **21** | **40** | **11** | **130** |
| **Sampling fraction** | 3.77 | 7.84 | 5.56 | 7.37 | 8.40 | 11.87 | 8.59 | 7.57 |
| **Overall weighting** | 26.5 | 12.7 | 18.0 | 13.5 | 11.9 | 8.5 | 11.6 |  |
| **Number of CEAs that used urine tests** | 0 | 4 | 10 | 19 | 21 | 11 | 6 | 71 |
| **Urine test weighting** | 0 | 38.3 | 18.0 | 15.7 | 11.9 | 30.6 | 21.3 |  |
