# Supplementary material for: Community burden of undiagnosed HIV infection among adolescents in Zimbabwe following primary healthcare-based provider-initiated HIV testing and counselling: A cross-sectional survey
Source: PLoS Med. 2017 Jul 25;14(7):e1002360. doi: 10.1371/journal.pmed.1002360 (PMC5526522; doi:10.1371/journal.pmed.1002360)
Supplement: S2 Table — (DOCX) [file pmed.1002360.s003.docx]

## Supplementary Table 2: Factors associated with HIV among all participants (N=5486)

| **Risk factor** |  | **HIV negative, n(%)** | **HIV positive, n(%)** | **Univariate** | | **Multivariate** | |
| --- | --- | --- | --- | --- | --- | --- | --- |
|  |  | **n=5345** | **n=141** | **OR (95% CI)** | **p** | **OR (95% CI)** | **p** |
| **Gender** | Male  Female | 2495 (46.7)  2850 (53.3) | 70 (50.7)  71 (50.4) | 1  0.84 (0.58, 1.21) | -  0.35 | 1  0.94 (0.63, 1.39) | -  0.75 |
| **Age group** | 8-12  13-17 | 2775 (51.9)  2570 (48.1) | 60 (42.6)  81 (57.5) | 1  1.44 (0.98, 2.11) | -  0.06 | 1  1.03 (0.71, 1.51) | -  0.87 |
| **Parent died** | No  Yes | 4490 (84.0)  855 (16.0) | 77 (54.6)  64 (45.4) | 1  4.11 (2.72, 6.20) | -  <0.01 | 1  3.24 (2.08, 5.04) | -  <0.01 |
| **Marital status** | Never married/lived together  Ever married/ lived together | 5293 (99.0)  52 (1.00) | 138 (97.9)  3 (2.1) | 1  1.36 (0.42, 4.42) | -  0.61 |  |  |
| **Education** | Normal/high grade for age  Low grade for age/never been to school | 3598 (67.3)  1747 (32.7) | 64 (45.4)  77 (54.6) | 1  2.57 (1.81, 3.66) | -  <0.01 | 1  2.22 (1.53, 3.21) | -  <0.01 |
| **Caregiver** | Parent  Not a parent | 3929 (73.5)  1416 (26.5) | 81 (57.5)  60 (42.6) | 1  1.89 (1.29, 2.77) | -  <0.01 | 1  1.20 (0.84, 1.72) | -  0.32 |
| **Share food with HIV+ child** | Comfortable  Uncomfortable | 4848 (90.9)  484 (9.1) | 136 (96.5)  5 (3.6) | 1  0.30 (0.12, 0.76) | -  0.01 | 1  0.28 (0.11, 0.73) | -  <0.01 |
| **Age of HH head** | <24 [reference group] | 108 (2.0) | 5 (3.6) | 1.34 (1.02, 1.77)* | 0.04 | 1.15 (0.88, 1.50)* | 0.30 |
|  | 25-39 | 1949 (36.5) | 39 (27.7) |  |  |  |  |
|  | 40-59 | 2580 (48.3) | 68 (48.2) |  |  |  |  |
|  | 60+ | 707 (13.2) | 29 (20.6) |  |  |  |  |
| **Education of HH head** | Secondary/further  None/primary | 4886 (91.4)  458 (8.6) | 127 (90.1)  14 (9.9) | 1  1.29 (0.53, 3.14) | -  0.58 |  |  |
| **HH monthly income** | No regular salary [reference group] | 2806 (52.5) | 86 (61.0) | 0.87 (0.75, 1.01)* | 0.07 | 0.94 (0.80, 1.11)* | 0.50 |
|  | USD 1-200 | 218 (4.1) | 5 (3.6) |  |  |  |  |
|  | USD 201-500 | 1440 (27.0) | 32 (22.7) |  |  |  |  |
|  | >USD 500 | 880 (16.5) | 18 (12.8) |  |  |  |  |
| **Hospital admission** | Never admitted  Ever admitted | 5050 (94.5)  295 (5.5) | 104 (73.8)  37 (26.2) | 1  5.86 (3.84, 8.95) | -  <0.01 | 1  5.94 (3.74, 9.42) | -  <0.01 |

* Odds ratio for trend
