# Supplementary material for: Community burden of undiagnosed HIV infection among adolescents in Zimbabwe following primary healthcare-based provider-initiated HIV testing and counselling: A cross-sectional survey
Source: PLoS Med. 2017 Jul 25;14(7):e1002360. doi: 10.1371/journal.pmed.1002360 (PMC5526522; doi:10.1371/journal.pmed.1002360)
Supplement: S3 Table — (DOCX) [file pmed.1002360.s004.docx]

## Supplementary Table 3: unweighted HIV prevalence and proportion with undiagnosed HIV

| Participants | Age group (years) | N | HIV positive | Crude HIV prevalence (95% CI) | Undiagnosed HIV | Crude prevalence of undiagnosed HIV (95% CI) |
| --- | --- | --- | --- | --- | --- | --- |
| All participants | **All** | **5486** | **140** | **2.55% (2.15-3.00%)** | **53** | **37.9% (29.8-46.4%)** |
|  | 8-12 | 2835 | 60 | 2.12% (1.62-2.72%) | 17 | 28.3% (17.5-41.4%) |
|  | 13-17 | 2651 | 80 | 3.02% (2.40-3.74%) | 36 | 45.0% (33.8-56.5%) |
| Only participants with urine test result | **All** | **2643** | **76** | **2.88% (2.27-3.59%)** | **25** | **32.9% (22.5-44.6%)** |
|  | 8-12 | 1401 | 36 | 2.57% (1.81-3.54%) | 10 | 27.8% (14.2-45.2%) |
|  | 13-17 | 1242 | 40 | 3.22% (2.31-4.36%) | 15 | 37.5% (22.7-54.2%) |
