# Supplementary material for: Community burden of undiagnosed HIV infection among adolescents in Zimbabwe following primary healthcare-based provider-initiated HIV testing and counselling: A cross-sectional survey
Source: PLoS Med. 2017 Jul 25;14(7):e1002360. doi: 10.1371/journal.pmed.1002360 (PMC5526522; doi:10.1371/journal.pmed.1002360)
Supplement: S1 Text — (PDF) [file pmed.1002360.s005.pdf]

1329301852

ZP01 BRTI -ZENITH HOUSEHOLD BASELINE FORM

SECTION 1: GENERAL QUESTIONS TO BE FILLED FOR EVERY HOUSEHOLD (HH)

SECTION1: GENERAL QUESTIONS TO BE FILLED FOR EVERY HOUSEHOLD (HH)			
**H00**	INTID	Interviewer's ID	☐☐
**H01**	HSEID	Household ID	☐
**H02**	BURB	Suburb Name	Budiriro ☐ Kuwadzana ☐ Dzivarasekwa ☐ Mufakose ☐ Highfield ☐ Glen Norah ☐ Glenview ☐
**H03**	CEA	Census enumeration area code	☐☐☐☐☐
**H04**	ADRS	Physical address	☐☐☐☐☐☐☐☐☐☐☐☐☐☐☐ ☐☐☐☐☐☐☐☐☐☐☐☐☐☐☐ ☐☐☐☐☐☐☐☐☐☐☐☐☐☐☐
**H05**	DATE	Date of Interview	☐☐ ☐☐ ☐☐☐☐
**H06a**	LATIT	Latitude	☐☐.☐☐ S
**H06b**	LONG	Longitude	☐☐.☐☐ E
**H06c**	ALT	Altitude	☐☐☐☐.☐☐ m
**H07a**	VISIT1	Visit 1	Complete ☐ Refused ☐ Part Complete ☐ Vacant HH ☐
**H07b**	VISIT2	Visit 2	Complete ☐ Refused ☐ Part Complete ☐ Vacant HH ☐
**H07c**	VISIT3	Visit 3	Complete ☐ Refused ☐ Part Complete ☐ Vacant HH ☐
**H06**	INTNME	Interviewee Name	☐☐☐☐☐☐☐☐☐☐☐☐☐☐☐
**H07**	HSURM	Head of household surname	☐☐☐☐☐☐☐☐☐☐☐☐☐☐☐

ZP01 BRTI -ZENITH HOUSEHOLD BASELINE FORM

Household Information: The head of the household should supply the following information OR in his/her absence, a representative with sufficient knowledge about the household and permission to answer in the place of the head of household

**H08**	**HHS**	How many people live in this HH?<br>Include all people who normally live and share meals in the HH<br>(*Mumba menyu munogara vanhu vangani?*<br>*Vanhu vanosanganisavese vanodya pamwe chete mumba muno)*	☐☐
**H09**	**OHHS**	How many people aged more than 17 years live in this HH<br>(Include all people who normally live and share meals in the HH)<br>(*Mumba menyu munogara vanhu vane makore anodarika 17 years vangani?)*	☐☐
**H10**	**OSEX**	Of those aged over 17 years, how many are male and how many female?<br>(*Kana pane vane makore anodarika 17 years,varume vangani nevakadzi vangani?)*	Male ☐☐<br>Female ☐☐
**H11**	**UHHS**	How many children aged under 8 years old live in this HH?<br>(*Mumba menyu munogara vanhu vane makore ari pasi pe 8 years vangani?)*	☐☐
**H12**	**YHHS**	How many children aged 8-17 years live in this HH<br>*Include all people who normally live and share meals in the HH*<br>(*Mumba menyu munogara vanhu vane makore ari pakati pe 8-17 years vangani?)*	☐☐
**H13**	**YSEX**	Of those aged 8-17 years , how may are male and how many are female?<br>*Include all people who normally live and share meals in the HH*<br>(*Kana pane vane makore aripakati pe 8-17 years, varume vangani nevakadzi vangani)*	Male ☐☐<br>Female ☐☐
**H14**	**SEL**	Select this household in prevalence survey?<br>**Select HH if any children aged 8-17 years live in HH**	Yes ☐ No ☐

If household not selected, thank respondent and leave

SECTION 2: QUESTIONS TO BE FILLED FOR SELECTED HOUSEHOLDS ONLY
Now I am going to ask you a few more questions about your household

**H15**	**HAGE**	How old is the HH head?<br>(*Muriritiri wemhuri ino anemakore mangani?)*	☐☐
**H16**	**HSCH**	What is the highest level of education the HH head has<br>(*Muriritiri wemhuri ino akadzidza kusvika papi?)*	Primary ☐ University ☐<br>Secondary ☐ Training College ☐<br>None ☐

ZP01 BRTI -ZENITH HOUSEHOLD BASELINE FORM

H17	OWN	Does the household own the dwelling?<br>(*Mhuri yenyu ndiyo muridzi wenzvimbo ino here?)*	Own Dwelling ☐<br>Rent Main Dwelling ☐<br>Rent Part of Dwelling/Lodger ☐<br>Use dwelling without Paying Rent ☐
H18	TYP	What section of the dwelling does the household reside in?<br>(*Mhuri yenyu inogara mumupanda upi weimba?)*	Full Main House ☐<br>Part of Main Dwelling ☐<br>Cottage/Out Building ☐<br>Part of Cotage/Outbuilding ☐
H19	STUC	What is the structure the household dwells in made of?<br>*(Imba yamunogara yakavakwa nei?)*	Brick under tile ☐<br>Brick under asbestos ☐<br>Brick under corrugated iron ☐<br>Wood Cabin ☐<br>Other ☐
H20	RMS	How many rooms is the household using?<br>(*Mhuri yenyu inoshandisa mipanda mingani paimba ino?)*	☐☐
H21	ASST	Does your HH own any of the following<br>(please cross (X) to all that apply)<br>*Car/Truck in working order<br>(*Mhuri yenyu ine zvinhu izvi here?)*	Fridge ☐<br>Car/Truck* ☐<br>Television ☐<br>Cellphone ☐
H25	SAL	How many people in the HH receive a regular salary? (By "regular salary" I mean money that is paid by an employer daily, weekly or monthly during the last 2 months or longer)<br>(*Mumhuri yenyu, mune vanhu vangani vanoshanda vachitambira mari (Apa ndiri kutaura muhoro wakawanikwa kupera kwemwedzi kana svondo mumwedzi miviri yapfuura)*	☐☐
H26	SALQ	What would you say the regular HH income would be per month?<br>(*Mari inotambirwa mumba ino pamwedzi ingaita marii pamwedzi?)*	Less than USD100 ☐<br>USD201-500 ☐<br>USD501-900 ☐<br>More than USD900 ☐<br>Dont know/dont want to say ☐

SECTION 3: HOUSEHOLD MORTALITY

I am now going to ask you for information on any member of the household who has passed away in the last 12 months

H29	DIED	Has any member of the household passed away in the last 12 months since - MONTH 2014/2015?<br>(*Mumwedzi gumi nemaviri apfuura, pane munhu mumhuri yenyu akashaya here - Mwedzi 2014/2015?)*	Yes ☐ No ☐

If no, move straight to SECTION 4. If yes, complete questions below and then move to SECTION 4

H30	NDD	How many members passed away in this HH<br>(*Kana pane akashaya, vangani vakashaya)*	☐☐

ZP01 BRTI -ZENITH HOUSEHOLD BASELINE FORM

		For each deceased	Person 1	Person 2	Person 3
**H31**	**DAGE**	What was the age of the deceased at the time of their death<br>*Aiva namakore mangani paakashaya*	☐☐ Yrs	☐☐ Yrs	☐☐ Yrs
**H32**	**DSEX**	What was the sex of the deceased?<br>*Aiva murume kana mukadzi here*	Male ☐<br>Female ☐	Male ☐<br>Female ☐	Male ☐<br>Female ☐
**H33**	**DCSE**	What was the cause of death of the deceased?<br>*Akashaya nei*	Trauma ☐<br>Other ☐	Trauma ☐<br>Other ☐	Trauma ☐<br>Other ☐
**H34**	**DLOC**	In which location did the deceased die?<br>*Akashayikira kupi*	Home(here) ☐<br>Hospital ☐<br>Rural Home ☐<br>Other ☐	Home(here) ☐<br>Hospital ☐<br>Rural Home ☐<br>Other ☐	Home(here) ☐<br>Hospital ☐<br>Rural Home ☐<br>Other ☐

SECTION 4: HIV AWARENESS

**H35**	**HIV**	Have you heard of HIV/AIDS?<br>Makambonzwa nezveHIV/AIDS here?	Yes ☐ No ☐
**H36**	**PLAY**	How comfortable would you be for a child in your HH to play with a child who is HIV-positive?<br>*Makasungunuka henyu here kuti mwana wenyu atambe nemwana wamunoziva kuti ane HIV*	Very comfortable ☐<br>A little uncomfortable ☐<br>Very uncomfortable, but allow it ☐<br>Would forbid it ☐
**H37**	**VST**	How comfortable would you be for a child who is HIV-positive to visit your HH?<br>*Makasungunuka henyu here kuti mushanyirwe nemwana wamunoziva kuti ane HIV*	Very comfortable ☐<br>A little uncomfortable ☐<br>Very uncomfortable, but allow it ☐<br>Would forbid it ☐
**H38**	**SHARE**	How comfortable would you be for a child in your HH to share food with a child who is HIV-positive?<br>*Makasungunuka henyu here kuti mudye pamwechete nemwana wamunoziva kuti ane HIV*	Very comfortable ☐<br>A little uncomfortable ☐<br>Very uncomfortable, but allow it ☐<br>Would forbid it ☐
**H39**	**OPT**	Ask respondent to pick a card to select randomisation option	Option 1(N) ☐ Option 2 (V) ☐ Option 3 (D) ☐

The questionnare is complete - thank the respondent
