# Supplementary material for: Community burden of undiagnosed HIV infection among adolescents in Zimbabwe following primary healthcare-based provider-initiated HIV testing and counselling: A cross-sectional survey
Source: PLoS Med. 2017 Jul 25;14(7):e1002360. doi: 10.1371/journal.pmed.1002360 (PMC5526522; doi:10.1371/journal.pmed.1002360)
Supplement: S2 Text — (PDF) [file pmed.1002360.s006.pdf]

ZP02 INDIVIDUAL QUESTIONNAIRE: PREVALENCE SURVEY

SECTION1: INFORMATION ABOUT THE INDIVIDUAL			
**I am going to ask you (child) a few more questions about yourself and your family. Ask adult respondent to answer if child unable to answer a question**			
**P01**	INTID	Interviewer's ID	☐☐
**P02**	STID	Study ID No	☐
**P03**	DATE	Date of Interview	☐☐ ☐☐ ☐☐☐☐
**P04**	DOB	Date of birth<br>*Wakaberekwa rinhi?*	☐☐ ☐☐ ☐☐☐☐
**P05**	AGE	Age in years<br>(Calculate or estimate age if not known)	☐☐
**P06**	SEX	Sex	Male ☐ Female ☐
**P38**	INTR	Interview result	Interviewed ☐<br>Refused ☐<br>Expected back in more than 2 weeks ☐<br>Not found after 2 visits ☐
**P07**	MALV	Is your mother alive?<br>*Mai vako vapenyu here?*	Yes Living in this HH ☐<br>Yes, But lives in another HH in Harare ☐<br>Yes, but lives in another city/rural area ☐<br>Yes, but lives in another country ☐<br>No ☐<br>Don't Know ☐
**P08**	FALV	Is your father alive?<br>*Baba vako vapenyu here?*	Yes Living in this HH ☐<br>Yes, But lives in another HH in Harare ☐<br>Yes, but lives in another city/rural area ☐<br>Yes, but lives in another country ☐<br>No ☐<br>Don't Know ☐
**P09**	MCARE	Who is your main caregiver?<br>(must be living in the same HH)<br>*Ndiani muchechengeti wako wauri kugara naye iyezvino?*	Biological mother or father ☐<br>Aunt/Uncle ☐<br>Grandparent ☐<br>Sibling ☐<br>Other Relation ☐<br>Non-relation ☐
**P10**	RES	Do you usually live and sleep in this household at least 4 nights a week?<br>Unowanza kugara nekurara mumhuri ino here [illegible]	Yes ☐ No ☐

ZP02 INDIVIDUAL QUESTIONNAIRE: PREVALENCE SURVEY

**P11**	PER	How long have you been living in this HH?<br><br>Wava nenguva yakadini uchigara mumhuri ino?	Years (if Living in HH for >12 months) ☐☐<br>months (if living in HH for < 12months) ☐☐<br>days (if living in HH for < 1 month) ☐☐
**P12**	ORES	How many HH have you live in since you were born (including this one)?<br>Imhuri ngani dzawakambogara nadzo tichiverenga mhuri ino	☐☐
**P13**	MAT	What is your current marital status<br><br>Wakawanikwa/Wakawana here?	Never Married ☐<br>Living together as if married ☐<br>Married ☐<br>Married but not living together ☐<br>Separated/Divorced ☐<br>Widowed ☐
**P14**	SCH	Are you currently going to School?<br><br>Uri kuenda kuchikoro here?	Yes ☐ No ☐
**P15**	LVL	What is the highest level of education you have completed?<br><br>Wakadzidza kusvika papi?	Never Been to School ☐<br>Primary ☐ write Grade ☐<br>Secondary ☐ write Form ☐<br>Higher ☐
**P16**	DSIB	Have you had a natural sibling (share the same mother) who died in the past?<br><br>Wakamboshaikirwa nehanzvadzi komana/sikana kana munin'ina wemudumbu mumwe here?	Yes ☐ No ☐

SECTION2: HEALTH AND DEVELOPMENT

Now I am going to ask you (child) a few more questions about your health. Ask adult respondent to answer if child unable to answer a question

**P17**	HLTH	How do you rate your general health?<br><br>Unofunga kuti utano hwako hwakamira sei?	☐ Excellent<br>☐ Good<br>☐ Fair<br>☐ Poor
**P18**	TB	Have you ever been treated for TB (mark yes if currently on treatment)<br><br>Wakamborapwa kana uri kurapwaTB here ?	Yes ☐ No ☐
**P19**	ADMT	Have you ever been admitted to hospital<br><br>Wakambogara muchipatara uchirwara here?	Yes ☐ No ☐

ZP02 INDIVIDUAL QUESTIONNAIRE: PREVALENCE SURVEY

**P20**	SKN	Have you been having recurring skin problems<br>*Unonetsekana nechirwere chemaronda kana mapundu/mhezi here?*	Yes ☐ No ☐
SECTION 3: HIV TESTING AND CARE			
I am now going to ask questions about HIV testing (questions to be addressed to the household adult respondent if appropriate)			
**P21**	HIVT	Has the child ever been tested for HIV?<br>*Mwana akamboongororwa kuti ane utachiona hweHIV here?*	Yes ☐ No ☐
**P22**	HIVD	What was the date of the last HIV test the child had?<br>*Akaongororwa rinhi (gore)?*	☐☐ ☐☐ ☐☐☐☐
**P23**	HIVP	Where was the HIV test done?<br><br>*Akaongororwa kupi?*	At a primary care clinic in Harare ☐<br>At a hospital ☐<br>Private clinic/Private doctor ☐<br>In community (e.g mobile testing) ☐<br>VCT Center (e.g New Start) ☐<br>Other ☐<br>Not tested previously ☐
**P24**	CLIN	If tested at a Primary care clinic in Harare, at which clinic was the test done?<br><br>*Kana akaongororwa pakiriniki, akaongororwa pakiriniki ipi?*	Budiriro ☐<br>Highfields ☐<br>Kuwadzana ☐<br>Mufakose ☐<br>Dzivaresekwa ☐<br>Glen View ☐<br>Glen Norah ☐<br>Other ☐<br>Not tested Previously ☐
**P25**	HIVK	Does the child know the HIV test result?<br>*Mwana anoziva zvakabuda paakaongororwa here?*	Yes ☐<br>No ☐<br>Not previously tested ☐
**P26**	HIVR	What was the HIV test result?<br>*Zvakanzi akamira sei?*	Positive ☐<br>Negative ☐<br>Not tested previously ☐
**P27**	PRF	Can you provide proof of the HIV test<br>*Munezvinoratidza kuti akaongororwa here?*	Yes ☐<br>No ☐<br>Not previously tested ☐
**P28**	TPRF	Type of proof provided?	Patient held clinical Record ☐<br>No proof of test available ☐<br>Testing slip ☐<br>Not tested previously ☐<br>Other ☐
**P29**	ACT	What actions were taken after the HIV test result?<br><br>Chii chakaitika shure kwokunge aongororwa?	Went for HIV care ☐<br>Did not act yet ☐<br>Don't want to answer ☐<br>Not applicable(test was negative/not tested) ☐

ZP02 INDIVIDUAL QUESTIONNAIRE: PREVALENCE SURVEY

**P30**	HIVC	If child is receiving HIV care, which clinic is now providing HIV care<br>**Beatrice Road, Wilkins, Harare Central or Parirenyatwa Hospital*<br><br>*Kana mwana achirapwa HIV, arikurapwa kupi?*	At a primary care clinic in Harare ☐<br>*At a hospital ☐<br>Private Clinic/Private doctor (eg Newlands) ☐<br>Clinic outside Harare ☐<br>Other ☐<br>Not applicable ☐
**P30**	SPEC	Specify if other	specify ________
**P31**	HIVCP	If receiving care at a Primary care clinic in Harare, at which clinic is he/she receiving care?<br>*Kana arikurapwa pakiriniki muHarare, arikurapwa pakiniki ipi?*	Budiriro ☐ Dzivaresekwa ☐<br>Highfields ☐ Glen View ☐<br>Kuwadzana ☐ Glen Norah ☐<br>Mufakose ☐ Other ☐
**P32**	ART	If child is HIV-positive and went for HIV care, is he/she taking ART drugs<br>*Kana mwana achirapwa HIV, arikunwa maARV here?*	Yes ☐ No ☐ Not applicable ☐
**P33**	COTR	If child is HIV-positive and went for HIV care, is he/she taking cotrimoxazole?<br>*Kana mwana achirapwa HIV, arikunwa ma cotrimoxazole here?*	Yes ☐ No ☐ Not applicable ☐
**P34**	HSEID	Household ID	☐
**P35**	OPT	Randomised to	Option 1(N) ☐ Option 2 (V) ☐ Option 3 (D) ☐

Collect specimen and discuss about diagnostic testing and randomisation if not tested already

**P36**	DIAG	ZPO4 form given	Yes ☐ No ☐

Comments
