# Supplementary material for: Community burden of undiagnosed HIV infection among adolescents in Zimbabwe following primary healthcare-based provider-initiated HIV testing and counselling: A cross-sectional survey
Source: PLoS Med. 2017 Jul 25;14(7):e1002360. doi: 10.1371/journal.pmed.1002360 (PMC5526522; doi:10.1371/journal.pmed.1002360)
Supplement: S3 Text — (PDF) [file pmed.1002360.s007.pdf]

7590480171

ZP03 BRTI -ZENITH ANONYMOUS HIV TEST FORM

Complete for every child aged 8-17 years at the selected household

PART A

**A00**	F INIT	Field Worker ID	
**A01**	HHID	Household ID	
**A02**	STUDYNO	Study No	
**A03**	DATEC	Date of Specimens Collection (dd/mm/yyyy)	
**A04**	SPEC1	Oral Mucosal Transudate collected	Yes ☐ No ☐
**A05**	TICOL	Time Oral Mucosal Transudate collected	:
**A06**	SPEC2	DBS collected	Yes ☐ No ☐ Refused ☐
**A06b**	SPEC3	Urine Collected	Yes ☐ No ☐ Refused ☐
**A07**	TIMEC	Time DBS/Urine Collected	:

PART B **FOR ORAL MUCOSAL TRANSUDATE TEST ONLY**

A08	DATER	Date OMT Specimen Received	
**A09**	TIR	Time OMT read	:
**A10**	RES01	Test Result	Positive ☐ NOT DONE ☐ Negative ☐ Indeterminate ☐

PART C **FOR DBS/URINE TEST ONLY - FOR LAB USE ONLY**

**A11**	LABNO	Lab Number	L
**A12**	DATER	Date DBS/Urine Received	
**A13**	TIMER	Time DBS/Urine Received	:
**A14**	DAREP	Date DBS/Urine Processed	
**A15**	TIMEP	Time DBS/Urine Processed	:
**A16**	RESD1	ARV Detected	Yes ☐ NOT DONE ☐ No ☐ Indeterminate ☐
