# Supplementary material for: Community burden of undiagnosed HIV infection among adolescents in Zimbabwe following primary healthcare-based provider-initiated HIV testing and counselling: A cross-sectional survey
Source: PLoS Med. 2017 Jul 25;14(7):e1002360. doi: 10.1371/journal.pmed.1002360 (PMC5526522; doi:10.1371/journal.pmed.1002360)
Supplement: S4 Text — (PDF) [file pmed.1002360.s008.pdf]

6318186890

ZP04 BRTI -ZENITH DIAGNOSTIC HIV TEST

**D00**	F INIT	Fieldworker ID	☐☐
**D16**	HHID	Household ID	☐
**D01**	STUDYNO	Study No	☐
**D02**	OPT	Randomised to	Option 1(N) ☐ Option 2 (V) ☐ Option 3 (D) ☐
CLINIC TEAM TO FILL BELOW			
**D03**	AMT	Amount	☐☐
**D04**	DAT	Date of HIV Test	☐☐ ☐☐ ☐☐☐☐
**D05**	RES	1ST TEST RESULT	Positive ☐ Negative ☐
**D06**	TICOL	2ND TEST RESULT IF FIRST TEST POSITIVE	Positive ☐ Negative ☐
**D07**	TIE	TIE-BREAKER TEST RESULT (if discordant)	Positive ☐ Negative ☐ NOT NEEDED ☐
**D08**	SPEC2	Final HIV test result	Positive ☐ Negative ☐
**D09**	CARD	Test Result written in patient notes	Yes ☐ No ☐
**D10**	WGT	Weight	☐ ☐.☐ kg
**D11**	HGT	Height	☐ ☐ ☐ cm
**D12**	CD4	CD4 count form filled and sample taken only if positive HIV	Yes Child Positive ☐ No Child Positive ☐ No Child Negative ☐
**D13**	AGE	Age (years)	☐☐
**D14**	SEX	Gender	Female ☐ Male ☐
**D15**	SER	Voucher Serial Number	☐☐☐☐
