# Supplementary material for: Community burden of undiagnosed HIV infection among adolescents in Zimbabwe following primary healthcare-based provider-initiated HIV testing and counselling: A cross-sectional survey
Source: PLoS Med. 2017 Jul 25;14(7):e1002360. doi: 10.1371/journal.pmed.1002360 (PMC5526522; doi:10.1371/journal.pmed.1002360)
Supplement: S5 Text — (DOC) [file pmed.1002360.s009.doc]

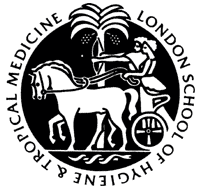
**ZENITH**

**Zimbabwe study for Enhancing Testing and Improving Treatment of HIV in Children**

***Sponsor* London School of Hygiene and Tropical Medicine, UK (LSHTM)**

***Funder* Wellcome Trust, UK**

***Principal Investigator* Dr Rashida A Ferrand (Clinical Senior Lecturer)**

***Host Institute* Biomedical Research and Training Institute, Zimbabwe**

***Local collaborators* Biomedical Research and Training Institute, Zimbabwe**

Prof Peter Mason

Prof Exnevia Gomo

**University of Zimbabwe Medical School**

Prof Kusum Nathoo

Dr Chiratidzo Ndhlovu

Dr Filda Bwakura

Dr Hilda Mujuru

**Ministry of Health and Child Welfare, HIV/TB Unit**

Dr Owen Mugurungi

Dr Angela Mushavi

**Department of City Health, Harare**

Dr Stanley Mungofa

**Elizabeth Glaser Pediatric AIDS Foundation, Harare**

Dr Agnes Mahomva

**Population Services International, Harare**

Dr Karin Hatzold

***International Collaborators* LSHTM, UK**

Prof Richard Hayes

Dr Liz Corbett

Joanna Busza

Dr Helen Weiss

**University of Oxford, UK**

Prof Sarah Rowland-Jones

| **Version 4.0 amended 01/06/2015 by RAF** |
| --- |
| Signature: Date: |

**TABLE OF CONTENTS**

[1 SUMMARY 6](#__RefHeading___Toc420919439)

[1.1 Introduction 6](#__RefHeading___Toc420919440)

[1.2 Aims 6](#__RefHeading___Toc420919441)

[1.3 Methodology 7](#__RefHeading___Toc420919442)

[1.4 Trial outcomes 7](#__RefHeading___Toc420919443)

[1.5 Study Timetable 8](#__RefHeading___Toc420919444)

[1.6 Intended use of results 8](#__RefHeading___Toc420919445)

[2 BACKGROUND 10](#__RefHeading___Toc420919446)

[2.1 Epidemiology of long-term survivors of mother-to-child transmission of HIV 10](#__RefHeading___Toc420919447)

[2.2 Complications of longstanding HIV infection 11](#__RefHeading___Toc420919448)

[2.3 HIV testing and care for older children 12](#__RefHeading___Toc420919449)

[2.4 Interventions to improve retention in HIV care 13](#__RefHeading___Toc420919450)

[3 OBJECTIVES AND STUDY DESIGN 15](#__RefHeading___Toc420919451)

[3.1 Study hypotheses 15](#__RefHeading___Toc420919452)

[3.2 Objectives 15](#__RefHeading___Toc420919453)

[3.3 Study Design 15](#__RefHeading___Toc420919454)

[3.4 Study outcomes 16](#__RefHeading___Toc420919455)

[3.4.1 PITC intervention 16](#__RefHeading___Toc420919456)

[3.4.2 Individual-randomised trial of household support 17](#__RefHeading___Toc420919457)

[3.4.3 Clinical cohort study 17](#__RefHeading___Toc420919458)

[4 STUDY POPULATION 18](#__RefHeading___Toc420919459)

[4.1 Study Suburbs 18](#__RefHeading___Toc420919460)

[4.2 HIV diagnosis and care services 18](#__RefHeading___Toc420919461)

[4.3 HIV Testing in Zimbabwe 19](#__RefHeading___Toc420919462)

[4.4 HIV Care Services 19](#__RefHeading___Toc420919463)

[4.5 Participant selection, inclusion and exclusion criteria 21](#__RefHeading___Toc420919464)

[5 METHODS AND PROCEDURES 23](#__RefHeading___Toc420919465)

[5.1 Routine PHC based PITC 23](#__RefHeading___Toc420919466)

[5.2 Implementation of Decentralised HIV care in PHCs 23](#__RefHeading___Toc420919467)

[5.3 Randomisation and investigator blinding 24](#__RefHeading___Toc420919468)

[5.4 Formative work 25](#__RefHeading___Toc420919469)

[6 HOUSEHOLD-LEVEL VLW SUPPORT 28](#__RefHeading___Toc420919470)

[6.1 Recruitment and training of LHW 28](#__RefHeading___Toc420919471)

[6.2 Duration of intervention 29](#__RefHeading___Toc420919472)

[6.3 Monitoring and Evaluation of the intervention 29](#__RefHeading___Toc420919473)

[7 HIV CLINICAL COHORT IN PHC 31](#__RefHeading___Toc420919474)

[7.1 Eligible participants 31](#__RefHeading___Toc420919475)

[7.2 Cohort study data collection 31](#__RefHeading___Toc420919476)

[7.3 PHC Patients not enrolled in cohort study 32](#__RefHeading___Toc420919477)

[7.4 Follow-up Schedule of cohort 32](#__RefHeading___Toc420919478)

[7.5 Assessment of growth 33](#__RefHeading___Toc420919479)

[7.6 Assessment of lung function 34](#__RefHeading___Toc420919480)

[7.7 Assessment of adherence 34](#__RefHeading___Toc420919481)

[7.8 Blood sampling 34](#__RefHeading___Toc420919482)

[8 POST-PITC PREVALENCE SURVEY FOR UNDIAGNOSED HIV INFECTION 35](#__RefHeading___Toc420919483)

[8.1 Sampling 35](#__RefHeading___Toc420919484)

[8.2 Participants 36](#__RefHeading___Toc420919485)

[8.3 Collection of data and biological samples 36](#__RefHeading___Toc420919486)

[9 OUTCOME EVALUATION 38](#__RefHeading___Toc420919487)

[9.1 Definitions 38](#__RefHeading___Toc420919488)

[9.2 Capture of Study Endpoints 38](#__RefHeading___Toc420919489)

[9.2.1 PITC 38](#__RefHeading___Toc420919490)

[9.2.2 Trial 38](#__RefHeading___Toc420919491)

[9.2.3 Post-PITC HIV Prevalence Survey 40](#__RefHeading___Toc420919492)

[9.2.4 Cohort Study 40](#__RefHeading___Toc420919493)

[9.3 Adverse Event Data Collection and Management 40](#__RefHeading___Toc420919494)

[10 DATA COLLECTION AND MANAGEMENT 41](#__RefHeading___Toc420919495)

[10.1 Data management 41](#__RefHeading___Toc420919496)

[10.2 Data capture forms and questionnaires 41](#__RefHeading___Toc420919497)

[10.3 Data security 41](#__RefHeading___Toc420919498)

[10.4 Quality assurance 41](#__RefHeading___Toc420919499)

[11 STATISTICAL CONSIDERATIONS 43](#__RefHeading___Toc420919500)

[11.1 Assumptions re. survival of HIV-infected children 43](#__RefHeading___Toc420919501)

[11.2 Sample size estimates 44](#__RefHeading___Toc420919502)

[11.2.1 Individual-randomised trial 44](#__RefHeading___Toc420919503)

[11.2.2 Post-Trial Prevalence Survey Sample Size Estimates 44](#__RefHeading___Toc420919504)

[11.2.3 Cohort Study 44](#__RefHeading___Toc420919505)

[12 LABORATORY METHODS 46](#__RefHeading___Toc420919506)

[12.1 Standard Operating Procedures 46](#__RefHeading___Toc420919507)

[12.2 Diagnostic and anonymised HIV testing 46](#__RefHeading___Toc420919508)

[12.3 CD4 count and viral load testing 46](#__RefHeading___Toc420919509)

[12.4 Processing and storage of blood specimens 46](#__RefHeading___Toc420919510)

[12.5 Safety issues 46](#__RefHeading___Toc420919511)

[13 ADMINISTRATION AND MONITORING 47](#__RefHeading___Toc420919512)

[13.1 Community Advisory Group 47](#__RefHeading___Toc420919513)

[13.2 Trial Steering Committee 47](#__RefHeading___Toc420919514)

[13.3 Good Clinical Practice 47](#__RefHeading___Toc420919515)

[13.4 Monitoring and audit 47](#__RefHeading___Toc420919516)

[14 ETHICAL CONSIDERATIONS 48](#__RefHeading___Toc420919517)

[14.1 Participant information leaflets 48](#__RefHeading___Toc420919518)

[14.2 Consent 48](#__RefHeading___Toc420919519)

[14.3 Disclosure of HIV status 49](#__RefHeading___Toc420919520)

[14.4 Ethics Committees providing review and approval 49](#__RefHeading___Toc420919521)

[14.5 Confidentiality 49](#__RefHeading___Toc420919522)

[14.6 Indemnity 49](#__RefHeading___Toc420919523)

[14.7 Trial registration 49](#__RefHeading___Toc420919524)

[15 STUDY TIME LINE 50](#__RefHeading___Toc420919525)

[16 DISSEMINATION 51](#__RefHeading___Toc420919526)

[16.1 Policy for sharing data 51](#__RefHeading___Toc420919527)

[16.2 Strategy for communication with user communities 51](#__RefHeading___Toc420919528)

[17 REFERENCES 52](#__RefHeading___Toc420919529)

[18 Appendices 58](#__RefHeading___Toc420919530)

[18.1 Map of Study sites 58](#__RefHeading___Toc420919531)

[18.2 PITC and HIV survey SOPs 58](#__RefHeading___Toc420919532)

[18.3 Formative Study Protocol and SOPs 58](#__RefHeading___Toc420919533)

[18.4 Trial SOPs 58](#__RefHeading___Toc420919534)

[18.5 VLW Intervention 58](#__RefHeading___Toc420919535)

[18.6 Cohort Study SOPs 58](#__RefHeading___Toc420919536)

[18.7 Laboratory SOPs 58](#__RefHeading___Toc420919537)

[18.8 Data handling and management 58](#__RefHeading___Toc420919538)

[18.9 Community Advisory Groups 58](#__RefHeading___Toc420919539)

[18.10 Other studies arising from the project 58](#__RefHeading___Toc420919540)

[18.11 Ethical approval and consent forms 58](#__RefHeading___Toc420919541)

[18.12 Needlestick injury policy 58](#__RefHeading___Toc420919542)

[18.13 Zimbabwe HIV testing and treatment Guidelines 58](#__RefHeading___Toc420919543)

[18.14 Study Forms 58](#__RefHeading___Toc420919544)

**ABBREVIATIONS**

ART Antiretroviral therapy

CBO Community-based organisation

DBS Dried Blood Spots

HCW Health care worker

FDC Fixed dose combination

IMCI Integrated Management of childhood Illnesses

MOHCW Ministry of Health and Child Welfare

MRCZ Medical Research Council of Zimbabwe

MTCT Mother-to-child HIV transmission

NRTI Nucleoside reverse transcriptase inhibitor

NtRTI Nucleotide reverse transcriptase inhibitor

PBMC Peripheral blood mononuclear cells

PHC Primary healthcare clinics

PI Protease inhibitor

PITC Provider-initiated testing and counselling

PSI Population Services International

VLW Voluntary lay health worker

# SUMMARY

## 1.1 Introduction

Southern Africa continues to bear a disproportionate share of the global AIDS burden, with the HIV prevalence over 10% in all the region’s countries. The natural history of vertically-acquired HIV is initially dominated by a very high risk of rapidly progressive disease, which led to the assumption in the early days of the HIV epidemic that survival to older childhood would be exceptional. However, it is now recognised that about a third of *all* HIV-infected infants can be expected to survive to 10 years even without prior treatment, due to slow-progressing disease. The combination of the high regional adult HIV prevalence rates during the 1990s, the lack of interventions to prevent mother-to-child HIV transmission at that time, and the unanticipated high likelihood of survival has led to substantial numbers of children presenting to healthcare services for the first time in older childhood with advanced HIV infection. Consequences of the under-estimation of this phase of the HIV epidemic include delayed HIV diagnosis and a lack of appropriate HIV care services for older children.

Late diagnosis in children is associated not only with a risk of the well-recognised HIV-associated infections, but also with a risk of chronic conditions that are not manifest in infants or adults, such as chronic lung and heart disease, growth failure and encephalopathy. Current recommendations for ART initiation in children over two years assume that treatment can be safely deferred until CD4 count/WHO Stage criteria (CD4 < 350cells/µl and/or WHO Stage 3/4 disease) are met. However, it is possible that delay in starting ART in slow progressors has an unacceptably high risk of serious chronic complications and suboptimal immune function.

Once ART is started, older children have a disproportionately high risk of poor adherence, with much worse treatment outcomes reported for adolescents than adults.^1-3^ With the tremendous pressures on health systems as a consequence of the HIV epidemic, there has been a move towards involvement of community health workers in supporting healthcare provision. As ART programmes scale up, lay workers may provide an effective and sustainable means of improving care outcomes in HIV-infected children.

## 1.2 Aims

The objective of the study is to investigate whether a package of services at primary care level will adequately meet the needs of vertically HIV-infected children and adolescents, or alternatively whether further decentralisation to provide community-level HIV testing and/or treatment support is required.

Specific aims are:

1. To investigate the impact of treatment support provided by lay volunteers to households with one or more HIV-infected children aged 6-15 years on adherence and retention into HIV care.
2. To investigate the effectiveness of routine facility-based HIV testing and counselling in reducing rates of undiagnosed HIV infection among children aged 6-15 years in the community, over a relatively short period of time.
3. To assess the effect of ART on lung function and growth in HIV-infected children aged 6-15 years.

## 1.3 Methodology

All children aged 6-15 years attending for acute care at primary health care clinics (PHC) in seven high-density suburbs of southwest Harare will be offered routine HIV counselling and testing during a two year period. The effectiveness of PHC-based routine PITC in providing high coverage of HIV diagnosis will be assessed through a prevalence survey for undiagnosed HIV infection in the 8-17 year age-group in the suburbs served by the PHCs. The seven PHCs will also provide decentralised HIV care, including antiretroviral therapy (ART) initiation.

Children who test HIV-positive through PITC at the PHCs will be recruited into an individual randomised trial of a voluntary lay worker (VLW)-delivered intervention to support households of HIV-infected children aged 6-15 years. Participants will be randomly allocated to either a) primary care based HIV care or b) primary care-based HIV care plus treatment support provided by VLWs, aim, aiming for equal number of control and intervention participants in each clinic. The intervention will be delivered over a eighteen month year period, the main outcomes being adherence to ART, and retention in care.

Children receiving HIV care at the PHCs including those recruited into the trial will be recruited into a clinical cohort study. Data from routine clinic visit (including height, weight, clinical symptoms, incident clinical events and adherence) will be collected. In addition, detailed growth monitoring, lung function testing and a psychosocial assessment (i.e. schooling, guardianship, and disclosure) will be carried out at pre-defined intervals. The rate of lung function decline and rates of pubertal development and growth will be compared by ART status, to investigate whether current ART initiation guidelines can prevent or delay progression of chronic lung disease and/or growth failure.

## 1.4 Trial outcomes

Comparison between intervention and control arms of:

- Proportion of children on ART with treatment failure defined as an HIV-1 viral load >400copies/ml at 12 months after starting ART or death in the first year of ART
- Proportion of children who miss two or more *routine* appointments by the end of two years of follow-up. This outcome has been chosen as studies have consistently shown that missed appointments are associated with worse health outcomes and poorer adherence to treatment.^4^

Other comparisons between arms will include:

1. all-cause mortality
2. numbers of and time to first hospital admission
3. numbers of unscheduled visits to PHC
4. proportion of children who have an average adherence rate <80% 12 months after starting ART
5. proportion of children who change to 2^nd^ line ART as a result of treatment failure

## 1.5 Study Timetable

This is a four year project starting in May 2012. Trial and cohort outcomes will be captured over a eighteen month period. PHC-based routine provider-initiated testing and counselling will be available for two years, following which prevalence of undiagnosed HIV infection in children in the community will be determined.

## 1.6 Intended use of results

The study results will be of international public health relevance; it will inform policy on decentralisation of testing and care services for HIV-infected children as well as on the need for revision of current guidelines for ART initiation for older children.

**Figure 1.1 ZENITH Project**

**ROUTINE PRIMARY CARE-BASED PITC**

PITC for children (6-15 years) & establishment of HIV care services

at 6 primary health clinics in 6 suburbs

**ZENITH OUTCOMES**

1. **Impact of PITC (prevalence survey)**

- Proportion of HIV-infected children who are still undiagnosed
- Overall HIV prevalence

1. **Impact of VLW intervention**

- Retention into care: proportion of children with ≥2 missed routine clinic visits
- Failure to suppress viral load on ART
- Morbidity and mortality

1. **Pre-ART care cohort**

- New onset and worsening lung function test abnormalities
- Pubertal development and growth
- HIV-associated infections and mortality

**POST-PITC HIV PREVALENCE SURVEY**

HIV prevalence survey after 2 years of PITC in 6000 children (aged 8-17 years)

**Follow-up of all children:**

- Missed clinic visits
- Death- interview family + hand-held records for cause of death
- Lung function, height, weight and head circumference, pubertal stage
- Adherence: self-report, pill-count
- School attendance and guardianship
- ART failure: HIV viral load and CD4 count
- Hospital admission and new diagnoses (hand-held records for cause)
- Identification of elite controllers

**CLINICAL COHORT STUDY**

Obtain consent to participate; PITC for siblings and guardians

Cotrimoxazole prophylaxis for all & start ART when eligible according to

National Guidelines

**Intervention: Standard HIV care + Household support intervention**

3 monthly clinic follow-up

+ scheduled home visits by VLW

**“Control”:**

**Standard HIV care**

3 monthly clinic follow-up in PHCs

**INDIVIDUAL RANDOMISED TRIAL**

Residing in study suburb randomised to:

Not residing in study suburb recruited into:

# BACKGROUND

## 2.1 Epidemiology of long-term survivors of mother-to-child transmission of HIV

HIV remains the leading cause of mortality in Southern Africa, which has the highest HIV prevalence of any global region. Regional adult HIV prevalence rates have exceeded 15% for the last 15 years, with even higher rates among pregnant women and subsequent high rates of mother-to-child HIV transmission (MTCT).^5^ The natural history of vertically-acquired HIV is initially dominated by a very high risk of rapidly progressive disease, with survival probabilities of only 50% at two years of age in African infant cohorts,^6^ leading to the assumption that survival to older childhood without treatment was exceptional.

As the HIV epidemic has matured, increasing numbers of older children with features of longstanding HIV infection have been presenting to healthcare services in Southern Africa.^7-8^ As observed in the West in the pre-ART era, recent studies show that a third of African infants do not have rapidly progressive disease and instead have at least as good a prognosis as HIV-infected adults in the absence of HIV care.^9-11^

Epidemiological data are compatible with a projected median survival estimate of 16 years among “slow-progressors” (no cohort has been followed for long enough to provide data-derived estimates), with 28% of *all* HIV-infected infants expected to survive to 10 years even in the absence of diagnosis and care (Figure 2.1).^12^

**Figure 2.1 Probability of survival to different ages following mother-to-child transmission of HIV infection**

**
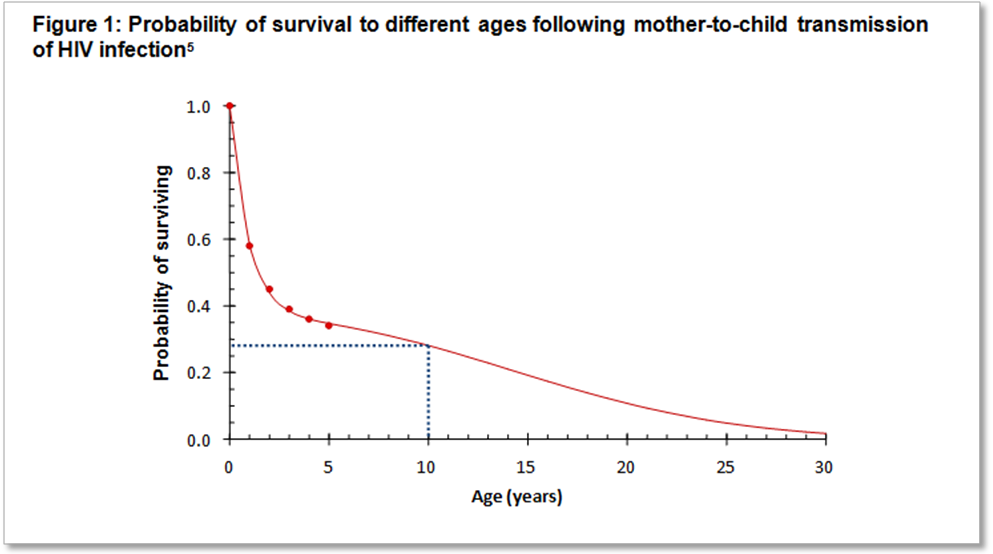
**

The combination of the high regional adult HIV prevalence rates during the 1990s, the lack of interventions to prevent MTCT (PMTCT) at that time, and the previously unexpectedly high likelihood of survival has led to a much bigger epidemic of “slow progressors” in the region than was anticipated. Consequences of the under-estimation of this phase of the HIV epidemic include:

- delayed diagnosis until presentation with unequivocal signs of advanced HIV with 75% of older HIV-infected children estimated to be living with HIV undiagnosed^7, 13-14^
- lack of development of appropriate health information and services for providing HIV care and advice on HIV prevention and sexual health for vertically-infected children as they reach adolescence
- a dearth of epidemiological studies, leading to a lack of understanding of the major causes of morbidity and mortality in older HIV-infected long-term survivors

Regionally, efforts to improve the implementation of PMTCT and the declining adult HIV epidemic will eventually lead to declines in the numbers of vertically-infected older children, but because of the intrinsic time-lag between infant exposure and reaching older childhood, long-term survivors can be expected to grow in numbers for at least a decade.^12^ Moreover, PMTCT coverage is incomplete (averaging 50% in the region), so that MTCT still occurs.^15-16^

## 2.2 Complications of longstanding HIV infection

Late diagnosis in children is associated not only with a risk of the well-recognised HIV-associated infections, but also with risk of chronic conditions that are not manifest in infants or adults. Nearly 50% of older HIV-infected children currently in care have life-threatening chronic lung disease, associated with severe respiratory insufficiency, hypoxia, and cor pulmonale.^17-18^

We have recently shown that constrictive obliterative bronchiolitis (OB) is the likely major underlying cause.^17^ OB affects the small airways, causing airflow limitation and hypoxic lung disease. It is difficult to diagnose (requiring high resolution CT scan, not chest radiography), and tends to be irreversible once established. OB can result from a number of insults, including post-adenovirus infection, and is an integral feature of the pathology of cystic fibrosis and rejection following lung transplantation, with some evidence for genetic predisposition to severe forms.^19-21^ OB has not been recognised in Western cohorts, suggesting either a geographical variability (e.g. due to more frequent exposure to respiratory tract viruses) or that early ART can prevent OB. In our series from Harare, however, OB was not restricted to patients with low CD4 counts and did not appear to respond to ART once established. However, this was a cross-sectional study and little is known about the natural history of this condition.

Pronounced stunting and pubertal delay are also common. Catch-up growth and pubertal development are likely to be age-dependent, with less good outcomes reported when ART is started in older children and adolescents.^22-23^ Untreated HIV infection is characterised by pronounced chronic immune activation,^24-26^ and inflammatory conditions appear unusually common in older children, including seronegative arthritis, cerebral vasculitis and uveitis.^7^ These conditions result in severe disability and functional impairment.

## 2.3 HIV testing and care for older children

Children face tremendous barriers to accessing HIV testing. Testing of <16 year olds requires consent of the legal guardian, who is often changing or absent. There are no free-standing HIV testing services available for children. Moreover, having been taught that once a child reached five years old they were unlikely to have vertically-acquired HIV infection, health workers have been reluctant to recommend HIV testing as part of the routine care of older children (since the default assumption then becomes sexual transmission, with the social and legal implications of inferred sexual abuse).^27^

Current recommendations for ART initiation in children over two years assume that treatment can be safely deferred until CD4 count/WHO Stage criteria (CD4 < 350cells/µl and/or WHO Stage 3/4 disease) are met.^28^ However, it is possible that delay in starting ART in slow progressors has an unacceptably high risk of serious consequences such as chronic lung disease, suboptimal immune function, stunting and compromised fertility.^23, 29^ In any event, ART tends to be started late in African programmes, which have a poor record of pre-ART care.^30-31^

Once ART is started, older children have a disproportionately high risk of poor adherence, with much worse treatment outcomes reported for adolescents than adults.^1, 3, 32-33^ Sub-optimal care-giving is among the potential factors (see Table 2.1) as treatment outcomes for chronic childhood illnesses are greatly influenced by guardians’ awareness and willingness to invest time and effort into accessing care. HIV-infected children may be at especially high risk of subtle forms of neglect because of maternal illness, as guardians tend to invest less in unhealthy children who are adopted or maternal orphans.^34-35^ Transition from paediatric to adult services (typically between ages 8-12 years in Africa) may also increase the risk of interruption in care.^36^

With the tremendous pressures on health systems as a consequence of the HIV epidemic, there has been a move towards involvement of community health workers in supporting healthcare provision.^49-50^ A wide range of community health worker models have been used in HIV/TB programs to provide care, support and advocacy outside the clinic setting.^51^ As ART programmes scale up, a community-based extension of health services may provide an effective and sustainable means of optimising care of HIV-infected children, particularly by strengthening engagement with healthcare services and supporting adherence to treatment.^42, 52-53^

**Table 2.1 Potential risk factors for non-adherence in older children**

| **Age** | Adolescence^37^ |
| --- | --- |
| **Disabling co-morbidity** | Chronic lung disease, neurocognitive impairment^17^ |
| **Adverse effects of treatment** | Real and perceived side-effects^38^ |
| **Poverty** | Competing priorities (food, school fees)^39^ |
| **Dependency and carer issues** | Orphanhood, carer-ill health, lack of supervision^40-41^ |
| **Distance from HIV clinic** | Journey time and transport costs^42-43^ |
| **Stigma** | Experienced or anticipated stigma^44^ |
| **Delayed disclosure of diagnosis** | To child^45-46^ |
| **Schooling** | Non-disclosure at school, reluctance to miss school, teacher & peer attitudes |
| **Health beliefs** | Disbelief in HIV as a biological disease entity^47-48^ |

## 2.4 Interventions to improve retention in HIV care

A large body of evidence demonstrates the importance of social support for long-term adherence to HIV treatment.^54-57^ In addition to physical and economic access to services, patients require an enabling environment to maintain retention in care.^58-59^ A recent analysis from South Africa, for example, found that over time, participating in support groups, having a treatment buddy, or receiving visits from a community health worker proved to be more significant predictors of treatment success than baseline health status or individual characteristics of patients enrolled in ART.^57^ Community support has been described as an informal “safety net” and shown to reduce attrition and improve adherence, most likely by reducing patients’ social isolation, increasing their understanding of the importance of treatment, and helping to tackle stigma.^60-61^

As a result, a wide range of community-based interventions have been introduced to strengthen social support and maximise retention in care.^62-65^ The use of community-based lay workers is one approach that has been shown to be effective for improving HIV treatment outcomes.^65^ Lay health workers are known by different terms including adherence support workers, ART aides, patient advocates, and community health volunteers (for those who are unpaid). Some programmes specify that they should be people living with HIV/AIDS themselves in order to share personal experiences and act as role models referred to as “expert patients” or peer support workers.^66-67^

Although the specific tasks differ, for the most part, lay health workers monitor ART patients’ attendance at scheduled clinic visits, and conduct home visits in order to identify and discuss barriers to treatment adherence, offer counselling and advice, assist with disclosure to family members, and remind patients of the importance of maintaining their clinical regimens. In some cases, lay health workers also accompany patients to clinics, facilitate support groups to bring ART patients together, and make referrals to any available benefits such as nutritional supplementation schemes, microfinance projects, or welfare grants.

Most programmes that have been evaluated have assessed their effect on the treatment success of adults. More recently, however, several studies have considered the role of community-based lay health workers in maximizing adherence and retention among HIV+ children, which relies on the commitment and motivation of their primary caregivers. ^56, 68^ Caregivers’ own physical and emotional health, understanding of HIV care, and access to supportive social networks will all affect their ability to sustain engagement with children’s clinical services.^55, 69^ In South Africa, a cohort study examined treatment outcomes among children enrolled in 47 public ART facilities. In households receiving home visits at which psychosocial support and adherence counseling were provided for children's caregivers, patient retention was 91.5% after 3 years of ART compared to 85.6% among those not receiving visits (p = 0.027). Mortality rates were also lower, at 3.7% vs. 8.0% (p = 0.060).^70^ In Kenya, a programme that adopted a “family centred” model that provided different forms of support to caregivers achieved survival rates of over 95% among children aged under 15 after 12 months on ART.^68^

Although it is difficult to generalise findings on lay health workers due to considerable heterogeneity across programmes, reviews of different models suggest that in terms of content, helping patients overcome denial, providing psychosocial support, and offering practical assistance (such as reminders of appointments) reduce loss-to-follow among both adults and children.^56, 60, 63^ The process of implementing community-based adherence support will also determine effectiveness, and the following components have been highlighted as critical to success: ensuring adequate levels of remuneration; providing good supervision, clear work guidelines and protocols; maintaining political support from and alignment with the formal health system; and offering on-going training to and motivating lay health workers.^65, 71^ Each programme needs to be adapted to its context and tailored to address locally-specific challenges of sustaining long-term retention in care, thus the ZENITH adherence support intervention will be based on formative research conducted in the study site, as well as draw on lessons learned from existing programmes.

# OBJECTIVES AND STUDY DESIGN

## 3.1 Study hypotheses

The starting hypotheses are, firstly, that optimised routine HIV testing and counselling of children aged >5 years attending acute primary care services will be sufficient to provide near-complete and timely (before the onset of severe immunosuppression or chronic complications) diagnosis of long-term survivors living in primary care clinic catchment areas within a relatively short period of time. This assumes that almost all HIV-infected children will have one or more consultations for minor illnesses during the two-year intervention period. Secondly, that decentralised HIV care services (to primary care level) are necessary but not sufficient to ensure good adherence and treatment outcomes, because of a constellation of risk factors for default in older survivors of mother-to-child transmission, notably their high risk of maternal orphanhood. Finally, that immediate commencement of ART regardless of CD4 count may be indicated in this age-group (as in infants), because of an otherwise unacceptably high risk of chronic lung disease.

## 3.2 Objectives

The broad aim of this study to investigate whether a package of services at primary care level will adequately meet the needs of vertically HIV-infected children and adolescents, or alternatively whether further decentralisation to provide community-level HIV testing and/or treatment support is required.

The study has three main components:

1. To investigate whether routine facility-based HIV testing and counselling of children aged 6-15 years attending acute primary care services provides high coverage of timely diagnosis of HIV in the clinic catchment areas.
2. To conduct a randomised control trial comparing a) optimised primary HIV care services alone with b) optimised primary HIV care services plus treatment support provided by VLWs to households with one or more HIV-infected older children.
3. To add serial lung function and anthropometry to the routine follow-up schedule of newly diagnosed children retained in HIV care, in order to investigate the safety of current deferred-start ART guidelines in this age-group (WHO stage 3/4 disease or CD4 count < 350cells/µl).

## 3.3 Study Design

The study will be conducted in the main PHCs of six suburbs in Southwest Harare. Each participating PHC will offer PITC routinely to 6-15 years olds over a two year period, a well as decentralised HIV care including ART initiation. Thus, children who test HIV-positive through PITC will be offered HIV care at the same PHC. At the end of two years of implementation of PITC, an anonymised HIV prevalence survey of 8-17 years olds from randomly selected dwellings in the suburbs served by the study PHCs will be conducted. The prevalence of *undiagnosed* HIV infection in the community following two years of PITC will provide an indication of the effectiveness of primary-care based routine HIV testing in providing high enough coverage of HIV diagnosis in this age-group (>80% of children diagnosed). The survey will also provide data on overall HIV prevalence by age in the considered age-group.

Children who test HIV-positive through PITC at the PHCs and reside in the study suburb where the clinic is located will be recruited into a trial of a VLW-delivered intervention to support to households of HIV-infected children aged 6-15 years. Stratified sampling will be used with randomisation of participants within clinics (1:1) to either a) primary care based HIV care or b) primary care-based HIV care plus treatment support provided by VLWs.

The intervention will be delivered over an eighteen month period, with formative work carried out to inform the design of the intervention. This will consist of qualitative interviews with care-givers of HIV-positive children attending for HIV care and health care providers (nurses, doctors, counsellors and pharmacists) at Harare Central Hospital as well as Community-based Organizations to understand the needs of households with HIV-infected children.

Children testing HIV positive at the PHCs including those recruited into the trial, will be recruited into a clinical cohort study. Data from routine clinic visit (including height, weight, clinical symptoms, incident clinical events and adherence) will be collected. In addition, detailed growth monitoring, lung function testing and a psychosocial assessment (i.e. schooling, guardianship, and disclosure) will be carried out at pre-defined intervals. The HIV care cohort will also provide a platform for immunological studies of slow-progression of HIV infection.

## 3.4 Study outcomes

### 3.4.1 PITC intervention

- Proportion of attendees who 1) accept HIV testing and 2) test HIV+ve following PITC
- Prevalence of undiagnosed HIV infection in the community two years post PITC implementation

### 3.4.2 Individual-randomised trial of household support

The unit of randomization will be an individual attending each clinic, and not households, however, the trial outcomes will be evaluated at the level of the clinic to cater for any clustering at clinic (suburb) level. The main differences between the two arms will be:

- Proportion on ART with treatment failure defined as an HIV-1 viral load >400copies/ml at 12 months after starting ART or death in the first year of ART
- Proportion who miss two or more *routine* appointments by the end of two years of follow-up

Other comparisons between arms will include:

1. all-cause mortality
2. numbers of and time to first hospital admission (defined as stay in hospital for ≥24 hours)
3. numbers of additional attendances (over and above routine follow-up) to PHC
4. proportion of children who have an average adherence rate <80% 12 months after starting ART
5. proportion of children who change to 2^nd^ line ART as defined by National Guidelines (due to reasons not related to toxicity, side-effects or starting TB treatment)

### 3.4.3 Clinical cohort study

The following outcomes will be determined for the clinical cohort:

- Mortality rate and cause of death
- Adherence rates
- Rate of failure of first-line ART
- Rates of incident opportunistic infections and tuberculosis
- Rates of hospital admission
- Rates of school attendance

In addition, the above outcomes and rate of lung function decline and rates of pubertal development and growth will be compared by ART status.

# STUDY POPULATION

## 4.1 Study Suburbs

Each high-density suburb is served by a main Primary Health Care Clinic (PHC) termed “polyclinic”, which provides acute and antenatal care services to the whole suburb. Six out of the ten high density suburbs in Western Harare were purposively selected on the basis that they have a have a high population of children ≤15 years and the polyclinics do not initiate ART to children in the target age-group. The chosen suburbs comprise approximately 194,885 children aged 6-15 years based on estimates from the Zimbabwe 2002 census.

## 4.2 HIV diagnosis and care services

Overall HIV prevalence in adults Zimbabwe has been declining and was estimated at 19.2% in 2011, 23.9% in 2010 and 30.6% in 2009 (National AIDS Council Annual Report, 2011). The adult HIV prevalence was estimated 19.1% in the study area during the 2008 DETECTB Household HIV Prevalence survey[[1]](#footnote-2). Estimates from the 2002 National Census indicated that there were approximately 120,182 children aged less than 16 years (mean 20,030, SD 4,409 children per suburb).

In a survey of 6 primary school located in the study suburbs, HIV prevalence among 6-14 year olds was estimated to be 2.7% (Table 4.1). Based on the DETECTB and School HIV Prevalence Survey estimates, the expected HIV prevalence among 6-14 year olds in the study suburbs was estimated to be approximately 14% of the adult prevalence, and thus estimated to range between 1.6% and 3.3%.

**Table 4.1 HIV prevalence among adults and primary school children in Study Suburbs**

| **Suburb** | **Adult HIV Prevalence (%)**  *(DETECTB Survey 2008)* | **6-14 yrs HIV Prevalence (%)**  *Primary school Survey 2010)* | **Expected HIV Prevalence in 6-14 yr olds (%)** |
| --- | --- | --- | --- |
| Suburb 1 | 18.43 | - | 2.58 |
| Suburb 2 | 18.75 | 2.89 | 2.62 |
| Suburb 3 | 15.34 | 2.09 | 2.15 |
| Suburb 4 | 23.75 | 3.78 | 3.32 |
| Suburb 5 | 18.46 | 2.05 | 2.58 |
| Suburb 6 | 19.99 | - | 2.80 |
| Suburb 7 | 16.59 | 1.79 | 1.66 |
| **Average** | **18.75** | **2.52** | **2.53** |

## 4.3 HIV Testing in Zimbabwe

HIV testing for adults (over the age of 16 years) is available through 1) client initiated testing provided by PSI Free Standing HIV Testing Services nationally and 2) Provider Initiated Testing and Counselling provided by primary and secondary level health facilities.

The Zimbabwean National Testing Guidelines recommend implementation of routine PITC at health-facilities for children and for adults. However, in practice, HIV testing is only offered to healthcare attendees on the basis of either symptoms that are consistent with HIV infection or client request.

Free-standing testing services do not usually offer HIV testing to minors. A person aged below 16 years wishing to have an HIV test, must be in the company of a parent/guardian. Before being offered testing, the parent/guardian (irrespective of whether they are biologically related or not) must be tested first and then give consent for the child to be tested. If the parent/guardian refuses then child will not be tested.

There was an increase in the number of clients who accessed HIV Testing and Counselling services from 837,789 in 2010 to 990,022 in 2011 (National AIDS Council Annual report 2011) (Table 4.2).

**Table 4.2: HIV testing and counselling (National AIDS Council Annual Report 2011)**

|  | **Age (yrs)** | **<15** | **15-19** | **20-24** | **25-29** | **30-49** | **50+** |
| --- | --- | --- | --- | --- | --- | --- | --- |
| **Clients tested** | Male | 37,848 | 40,980 | 67,995 | 78,741 | 148,405 | 49,031 |
|  | Female | 35,402 | 64,374 | 107,777 | 111,130 | 189,871 | 59,250 |
|  | Total | 73,250 | 105,354 | 175,772 | 189,871 | 338,276 | 108,281 |
| **Clients testing HIV Positive** | Male | 7,025 | 2,185 | 5,364 | 1,015 | 42,324 | 10,310 |
|  | Female | 6,142 | 5,070 | 15,891 | 24,343 | 50,691 | 9,690 |
|  | Total | 13,167 | 7,255 | 21,255 | 36,358 | 93,015 | 20,000 |
| **% HIV Positive** |  | 17.98 | 6.89 | 12.09 | 19.15 | 27.50 | 18.47 |

## 4.4 HIV Care Services

The south-western high density suburbs are serviced by two infectious disease hospitals (Wilkins Hospital and Beatrice Road Infectious Disease Hospital) at which all children under 16 years who test HIV positive are assessed and initiated on ART if eligible. Patients who are diagnosed following admission to the two central hospitals (Parirenyatwa or Harare Central Hospitals) may elect to receive their care at the HIV clinics at these two hospitals.

ART is initiated at Hospital HIV outpatient services but HIV treatment is decentralised to primary care once patients are stable on treatment. Thus, patients aged over 10 years are monitored for a period of three months after initiation of ART, and then they are transferred to their nearest Primary Health Care Clinic for continuation of treatment. Children aged below 10 years usually continue to receive ART at secondary care services.

For those not ready for ART initiation, there is no formal pre-ART care at present, although all HIV-infected patients are provided with life-long cotrimoxazole obtained from PHCs and are supposed to have twice yearly CD4 counts.

The treatment programme is well-functioning and there have been no major stock-outs of paediatric or adult formulations of ART drugs in Zimbabwe during 2011. Apart from the National AIDS Trust Fund, the country has been getting support from different partners for the ART programme. Most of the paediatric formulations are funded by Clinton Health Access Initiative (CHAI) Zimbabwe. The figure 4.1 shows the relative contributions by the major ART funding partners in 2011.

**Figure 4.1 Total ART patient support by funding partners 2011**


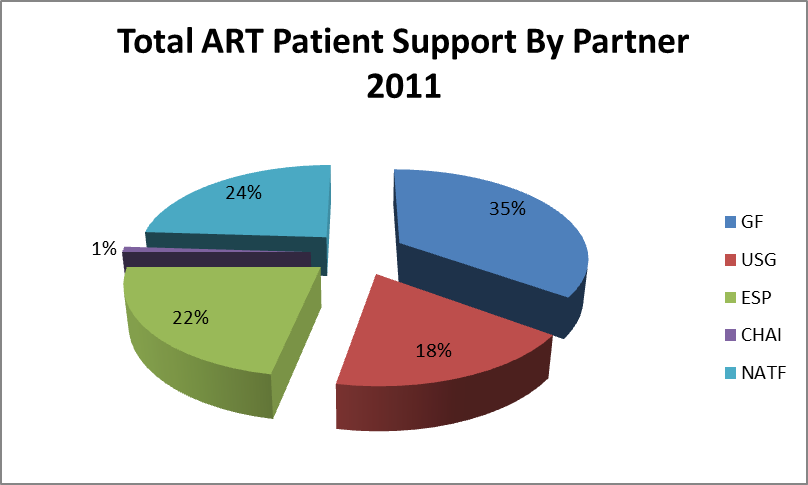


***Source: MOHCW 2011 ART Update***

A total of 463,361 clients were on ART in Zimbabwe by end 2011, out of the 593,000 in need of ART, with a 35% increase in coverage from 2010 to 2011. In terms of national coverage, Zimbabwe is now at 78.1% of those who urgently require ART. The patient survival rate for 2011 was 78% and 69% for 12 months and 24 months respectively.

In 2010 Harare City Health Clinics registered approximately 2,000 new HIV patients aged between 5-14years and 45% of them were from the South-Western High Density Suburbs. (HARARE CITY HEALTH Annual Report 2010)

**Table 4.3 Data on ART coverage 2011 (National ART programme)**

|  | **ZIMBABWE** | | **HARARE only** | | **STUDY SUBURBS only** | |
| --- | --- | --- | --- | --- | --- | --- |
|  | Children (<15years) | Adults | Children (<15years) | Adults | Children (<15years) | Adults |
| **Eligible for ART** | 2,006 | 19,934 | 96 | 1,750 | 11 | 167 |
| **Ever Initiated** | 44,877 | 477,706 | 8,963 | 97,525 | 1,821 | 9,859 |
| **Transferred Out** | 80 | 1,644 | 7 | 945 | 5 | 27 |
| **Transferred In** | 168 | 1,366 | 9 | 530 | 1 | 44 |
| **Currently On 1^st^ Line ART** | 40,273 | 415,991 | 8,793 | 75,028 | 1,821 | 3,155 |
| **Currently On 2^nd^ Line ART** | 332 | 6,765 | 150 | 4,199 | 62 | 99 |
| **Lost** | 247 | 870 | 61 | 136 | 0 | 14 |
| **Deceased** | 52 | 564 | 5 | 39 | 0 | 4 |

First and second-line treatment options and fixed-dose combinations (FDCs) with scored tablets are available for children. National ART Guidelines criteria (from May 2010) for starting ART in children are summarised below:

1. Paediatric WHO Clinical Stage 3 or 4 disease, irrespective of CD4 count or CD4%
2. Immunological criteria:

| **Immunological marker** | **Age-specific criteria for initiating ART** | | |
| --- | --- | --- | --- |
|  | **Infant ≤24 months** | **>24-60 months** | **≥5 years** |
| CD4 % | Treat all irrespective of CD4 count/ CD4% | <25% | N/A |
| CD4 count |  | <750cells/µl | <350cells/µl (as for adults) |

## 4.5 Participant selection, inclusion and exclusion criteria

The inclusion criteria for participation in the trial will be:

- age between 6 years (day of their 6^th^ birthday and older) and 15 years (a day before their 16^th^ birthday)
- test HIV-positive following PITC in the PHC
- willing to have their HIV care at the PHC
- usual residence in the study suburb where the PHC is located
- informed consent by parent and assent from child to participate

The exclusion criteria for participation in the trial will be:

- age <6 years or ≥16 years
- usual residence outside the study suburb where the PHC is located
- previously tested HIV+ positive and receiving care (clinical or psychosocial)
- not planning to access HIV care at the PHC

All participants enrolled into the trial will automatically be offered enrolment into the cohort study. Additionally, any children aged 6-15 years who test HIV-positive following PITC after enrolment for the trial is completed, will also be approached for recruitment into the clinical cohort study.

# METHODS AND PROCEDURES

## 5.1 Routine PHC based PITC

All children aged 6-15 years attending for acute care at primary health care clinics (PHC) in seven of the 10 high density suburbs of southwest Harare (Highfield, Glen View, Budiriro, Kuwadzana, Mufakose, Dzivarasekwa, Glen Norah) will be offered routine provider-initiated testing and counselling (PITC) during a two year period. Training on HIV testing and counselling of children will be provided through PSI.

A research assistant will be based at the clinic and will identify children eligible for PITC and refer them for HIV testing to PHC counsellors/nurses. After clinic registration, the standard approach to PITC developed by the Ministry of Health and Child Welfare will be used. All eligible clinic attendees will be given brief standard pre-test counselling followed by an offer of HIV testing and post-test counselling. Guardian consent will be required for children to undergo HIV testing. Children attending without a guardian will be given information about HIV testing and encouraged to re-attend with a guardian for HIV testing.

HIV test results will be given to the child and their guardian at the same clinic visit, with age-appropriate explanation of HIV test results given to the child. Children who test positive will be offered enrolment into HIV care at the PHC where they test. Biological parents, guardians and natural siblings of children who test HIV-positive will be offered HIV testing. Attendees who decline HIV testing will be assessed for their presenting health problem by the clinic nurse. PITC outcome will be recorded as accepted/ absent guardian/ testing for subject declined /testing for guardian declined.

HIV testing kits will be provided by the National HIV Program. The research assistants will ensure that a supply of testing kits is always available at the PHCs. Diagnostic HIV testing will be carried out using two HIV rapid tests (Abbott Determine™ and SD Bioline™) run in parallel. Discordant specimens will be resolved using a third rapid HIV test (INSTI™).

## 5.2 Implementation of Decentralised HIV care in PHCs

Nurse-led HIV care services for children will be established in the six PHCs that offer PITC. A designated nurse from each of the six PHCs will be invited to training course on paediatric HIV care at first-level facilities. The course will integrate HIV care with the IMCI clinical guidelines, as well as additional training on adolescent HIV care issues.

HIV care services at the PHC will include cotrimoxazole prophylaxis, routine follow-up visits, management of intercurrent illness as done within the routine primary health system (+emergency visits and referral to secondary care if indicated), disclosure and adherence counselling.

ART regimens and starting criteria will be dictated by National policy and practice, with drugs provided by the National ART Programme. Current National ART guidelines are summarised below:

| **Nevirapine-Exposed** | **Non-exposed** |
| --- | --- |
| ***First-line Treatment*** | |
| Stavudine+ lamivudine+ PI* | Stavudine+ lamivudine+ nevirapine |
| ***Alternative First-line Treatment*** | |
| Zidovudine +lamivudine + PI* | Zidovudine + lamivudine + nevirapine |

*Note: HIV-infected infants exposed to nevirapine (NVP) through infant or maternal treatment or prophylaxis, especially single-dose nevirapine, may have viral resistance*

**Current PI used is lopinavir/ritonavir*

*Abacavir and didanosine available as second-line drugs, in combination with PI or NVP (changing to the drug class that hasn’t been used in the first line regimen)*

At each follow-up visit, a standard symptom screen will be carried out and incident clinical events, adherence, height and weight recorded. At each visit, a prescription of ART will be given, with dose adjustment as required. Those starting ART will be seen every two weekly for the first month, monthly for the next two months, and three-monthly thereafter. Those not taking ART will be followed up at two weekly for the first month and three monthly thereafter.

Routine HIV care will be provided by PHC nurses. The research team will consist of a qualified nurse and a research assistant, with each team responsible for two PHCs. The research team will be responsible for identifying attendees eligible for PITC, supporting HIV care provision and collection of clinical cohort data. The study physician will be responsible for all 6 PHCs and will consult at the PHCs every two weeks and supervise the PHC HIV care providers, provide training updates and see complex cases. Ongoing mentoring for primary care nurses will be provided by City health doctors who consult at PHCs on a weekly basis.

## 5.3 Randomisation and investigator blinding

Eligible participants who reside in the study suburb where the PHC is located will be randomly allocated to either standard HIV care or to standard care + the support intervention (1:1 in each clinic). If the child resides outside Harare, he/she will be offered standard clinic care but will be excluded from the trial and the cohort study. All children aged 6-15 years registering for HIV care at the clinic and resident in Harare will be invited to participate in the cohort study, even if they refuse participation in the trial. The PI will be blinded to the information on the trial arm each participant is allocated to until the end of the trial follow-up.

**5.3.1 Randomisation Procedure**

Randomisation will use proportional stratified random sampling using computer generated pseudo –random numbers. The size of each strata will be proportionate to the population size of children aged 6-15 years who are serviced by the clinic (based on Census 2002 data) and are expected to be HIV positive (based on extrapolations from previous surveys in the same study suburbs)

- Stratification will be according to the recruitment clinic (6 clinics)
- The sampling fraction will be 0.175 to ensure that each clinic is allocated study numbers proportional to the expected population of children aged between 6-15 years who test HIV positive (sample which is proportional to size)
- Random allocation to either standard HIV care or to standard care + the support intervention of 1:1 at each clinic will be done, thereby each study number allocated to the clinic will be randomly allocated to either standard HIV care or to standard care + the support intervention
- As the children are recruited into the study, a study number will be allocated consecutively and the appropriate study arm linked to the number.

In the event that the child refuses to participate in the trial and/or is recruited in the cohort only a different study number will be allocated.

**Table 5.1: Expected sample size by suburb**

| **Stratum** | **Population Size**  **(2002 Census)** | **Estimated HIV Prevalence** | **Population Size (Expected HIV+ve)** | **Sampling Fraction** | **Final Sample Size** |
| --- | --- | --- | --- | --- | --- |
| PHC1 | 14519 | 2.58 | 375 | 0.175 | 32 |
| PHC2 | 24695 | 2.62 | 648 | 0.175 | 37 |
| PHC3 | 23036 | 2.15 | 494 | 0.175 | 64 |
| PHC4 | 23789 | 3.32 | 790 | 0.175 | 49 |
| PHC5 | 18556 | 2.58 | 479 | 0.175 | 78 |
| PHC6 | 15587 | 2.80 | 436 | 0.175 | 47 |
| PHC7 | 19203 | 1.66 | 318 | 0.175 | 43 |

## 5.4 Formative work

Formative research will consist of a short qualitative study to explore key local facilitators and barriers experienced by primary caregivers of HIV+ children aged 6-15 years in seeking to optimise their access to, retention in, and adherence to HIV treatment and care. It will provide data specifically for the design of the community-based adherence support intervention, and will build on existing research conducted in Zimbabwe on the contextual and social determinants of treatment adherence among paediatric HIV patients. The research question is "What local social and contextual factors enable children’s retention in HIV care and how might these be strengthened?"

The objectives of the formative research are to:

1. Describe HIV+ children’s caregivers’ perspectives on their experiences of caring for children requiring chronic HIV care, including challenges confronted related to maintaining appointments, ensuring treatment adherence, and supporting well-being more generally
2. Identify the specific material, symbolic and relational factors that enable HIV-infected children’s retention in care, and the local norms and social networks that either facilitate or hinder these factors.
3. Elicit the health providers’ views on how children, caregivers, other family members and communities engage with care, and their suggestions for how support can be provided at community level but remain linked to facilities.
4. Identify, map and obtain information on existing community-based initiatives that provide adherence support or similar outreach to families of HIV+ children.

Fieldwork will be conducted in the catchment area of Harare Hospital and take place in October 2012. Data will be collected from three types of respondents: (1) Harare Hospital health care providers, including those involved in clinical care and counselling/outreach; (2) Primary caregivers of HIV+ children aged 6-15 who have been enrolled in HIV care at Harare Hospital for at least 6 months; and (3) representatives or key informants from any existing community based organisations (CBO) engaged in providing adherence support or related services (such as distribution of food supplements, or home based care providers).

First, focus group discussions will be held with health care providers (both clinical and not), to identify the main issues relating to current provision of care to HIV+ children, and their perceptions of the unique challenges faced by the children’s primary caregivers, extended family members, and wider communities. The group discussions will also introduce the study to providers and familiarise them with the study fieldworkers, who will be collecting data regularly at the hospital.

Caregivers will then be recruited into the study for semi-structured interviews. They will be sampled to reflect diversity according to their relationship to the child(ren) for whom they care, and background characteristics including age, sex, and residence. Interviews will be conducted at the hospital and will address caregivers’ experiences of supporting HIV+ children in health-seeking, diagnosis, disclosure (if applicable), attending regular appointments, undertaking medical tests, taking medication, and positive living more broadly (mitigation of stigma, relationships at school, nutrition, psychosocial care, etc.)

Both providers and caregivers will be asked about existing community-based support programmes such as food supplementation, support groups, home based care visits, etc., and if any of these prove relevant, they will be mapped according to location, and services provided. Key informants will then be sought from each organisation and invited to a brief interview to determine what adherence support mechanisms already function in local communities, which appear to be successful, and why.

Analysis will use thematic content analysis and be driven by conceptual frameworks already developed in research conducted on barriers and facilitators to retention in care and treatment adherence. In particular, enabling conditions within family, community, and institutional structures will be extracted from the data, as well as any suggestions for what might work to increase sustained participation in care; these will be analysed with reference to evidence from the literature on the feasibility and effectiveness of different adherence support intervention components.

# HOUSEHOLD-LEVEL VLW SUPPORT

Community-based adherence support will be provided to families enrolled in the ZENITH trial by Voluntary Lay Workers (VLW) who will be recruited and trained to offer psychosocial counselling, practical advice, and links to appropriate referrals. The exact structure of the intervention, including guidelines and protocols specifying the nature and frequency of activities provided by VLWs, will be determined following the formative research described in Section 5.4, which will identify locally important facilitators and barriers experienced by the primary caregivers of HIV+ children. However, the LHW programme will centre around a series of home visits to (1) assess each enrolled family’s circumstances and needs; (2) identify feasible measures that caregivers might put in place to maximise engagement with care; (3) offer both general support and tailored assistance, following standardised guidelines; and (4) refer families to locally available organisations offering additional support services. Finally, LHW will serve as a link to the HIV treatment services by reminding caregivers of scheduled appointments, and following-up with clinic staff to check families’ attendance and identify emerging concerns that could lead to attrition from care.

## 6.1 Recruitment and training of LHW

Existing research on different models of community-based adherence support suggest that formalised management procedures, transparent employment policy, and clear terms of reference all contribute to job satisfaction and sustainability in lay health worker programmes. We will thus work through existing community based organisations to identify suitable candidates.

Eligibility criteria will include residence in the local community for at least 5 years, functionally literate (able to read and fill in rudimentary record-keeping forms, basic comprehension of literature such as health pamphlets circulated to clinic patients), willingness to travel on foot between households and regularly visit the local clinic, and ability to demonstrate experience of nurturing others (including caring for their own or others’ children, sick or elderly family members, or previous work experience in a caring capacity i.e. nurse, midwife, social worker). LHW workers will be contracted on a probationary period for two months (including the first two weeks of training) and their contract confirmed after a “refresher” workshop held at the end of the probationary period.

Training will be conducted over two weeks, followed by a further two weeks of intensive on-the-job supervision. Training will cover basic information related to HIV transmission, prevention, treatment regimens, care needs, and orientate LHW on common challenges families face in initiating treatment and sustaining adherence and retention in care. It will emphasise the importance of a family-centred strength-based case management approach, and explain the protocols developed for each scheduled home visit. The LHW will be taught how to address each family’s needs, provide information and support related to treatment adherence, including managing side-effects, encourage testing among other family members, encourage testing among other family member, and facilitate discussion around age-appropriate disclosure of HIV status to the child, and to other family and community members, where feasible.

Training will involve a mix of familiarisation with project guidelines, illustration through examples and case studies, role-plays, and participatory activities. During the intensive 2-week supervision period following the training, each LHW will be “shadowed” for a day as they conduct visits and contacted by phone at the end of each day to debrief and discuss any questions or uncertainties. Finally, after two months, a 2-day refresher workshop will be held so that LHW can discuss concerns, share initial experiences and problems, and work together as a team to seek solutions to any challenges confronted in their work. Additional 1-day workshops will be held every 6-9 months in order to maintain lesson-learning across the programme, build team unity, address any difficulties emerging during the programme, and ensure LHW receive up-to-date information and guidance related to the treatment component of the intervention.

## 6.2 Duration of intervention

The LHW intervention will be implemented over eighteen months. As new patients are enrolled in the clinic, their permission will be requested for referral to a LHW for an initial home visit. LHW will conduct a standardised assessment and describe the support and referrals provided through the project; new families enrolled in the study clinics will be visited following the same schedule as clinic appointments, namely once a fortnight for the first month, every 4 weeks in the following 2 months, and subsequently every 3-months. Children who are not yet eligible for ART will receive the same schedule of home visits, to encourage retention in care during the routine monitoring phase. Once they become eligible and initiate ART, the first 3 home visits will be repeated to deliver the activities related directly to treatment issues.

## 6.3 Monitoring and Evaluation of the intervention

In addition to the outcome evaluation comparing retention rates and clinical results between the intervention and control arms of the study, a process evaluation will be conducted during the LHW programme implementation. A monitoring and evaluation framework (see Appendix 18.5) will be developed during finalisation of the intervention design, and is likely to include documentation of activities (ease of recruiting LHW, completion rates for training, retention in programme, geographical coverage); compilation of routine statistics (client load per LHW, frequency of household visits, service mix provided, accompanied visits to health facilities); qualitative data collection to assess the quality of programme delivery (interviews with LHW, participatory evaluation exercises for client households, interviews with enrolled family members); and recording and analysis of any external events that could influence the intervention’s ability to achieve its aims (political instability, adverse weather conditions or catastrophic events, changes in relevant health or social policies).

# HIV CLINICAL COHORT IN PHC

## 7.1 Eligible participants

Children aged 6-15 years who are test HIV positive at the seven study clinics and reside in Harare will be eligible for recruitment into the cohort study, regardless of whether they are eligible for recruitment into the randomised trial. Patients aged 6-15 years who are already on ART and accessing care at a secondary health facility will be offered HIV care at the study PHC they attend, but will not be eligible for recruitment into the cohort study.

## 7.2 Cohort study data collection

Patients who test HIV positive and choose to register for HIV care at the PHC will be invited for an initial assessment within a week of diagnosis. The assessment will include:

- Record of social history: Current household structure, family history, type of guardianship, knowledge of HIV status, schooling
- Review of clinical history: HIV testing history, past infections including TB, other significant illness, hospitalisations, HIV testing history
- Clinical assessment: standardised symptom screen, WHO clinical stage
- Examination: standardised clinical examination, growth assessment, lung function assessment
- Tests: CD4 count, blood sample store

Patients will be referred for counselling and started on cotrimoxazole prophylaxis, and followed up two weeks later for assessment of ART eligibility (according to National Guidelines).

Those who are not eligible for ART will be followed up a month after the initial assessment, and then on a three monthly basis. At each visit, patients will have a brief symptom screen and undergo measurement of height and weight. Cotrimoxazole will be given and patients will have brief counselling. If participants start ART during the follow-up period, they will require an additional visit 2 and 4 weeks later, before reverting back to their follow-up schedule.

Those who are eligible for ART will be referred for counselling and started on the recommended first-line treatment. They will be then followed up at 2 weeks, and then monthly for 2 months and three monthly thereafter. At each visit, they will have a brief symptom screen, assessment of adherence, and measurement of height and weight. ART dose will be checked at each visit and adjusted according to weight.

For all participants, a CD4 count, growth and lung function assessment will be performed every 6 months, and psychosocial assessment (knowledge of HIV diagnosis, guardianship, schooling, disclosure to others) and pubertal staging carried out annually.

Participants who miss scheduled appointments will be offered alternative appointments for clinical review at the next available date. All participants will be followed up for 72 weeks. Participants recruited earlier in the study will continue to receive 3 monthly cohort follow-up until the study ends. At the end of the follow-up period, all participants will continue to receive their routine HIV care at the respective PHCs.

## 7.3 PHC Patients not enrolled in cohort study

Patients who register for HIV care at the PHC, but decline consent to participation into the cohort study will be receive the same level of care as participants enrolled into the clinical cohort. However, the following will not be carried out:

- Assessment of lung function
- Detailed growth and pubertal staging
- Viral load testing one year after starting ART
- Blood sampling for immunogenetic studies
- Detailed psychosocial and adherence assessment

## 7.4 Follow-up Schedule of cohort

**Table 7.1 Follow-up schedule for cohort participants who are not yet on ART**

|  | **Week** | | | | | | | | | | |
| --- | --- | --- | --- | --- | --- | --- | --- | --- | --- | --- | --- |
|  | **0** | **2** | **4** | **8** | **12** | **24** | **36** | **48** | **60** | **72** |  |
| Nurse visit^1^ |  | ✓ | ✓ | ✓ | ✓ | ✓ | ✓ | ✓ | ✓ | ✓ |  |
| Weight/Height | ✓ | ✓ | ✓ | ✓ | ✓ | ✓ | ✓ | ✓ | ✓ | ✓ |  |
| HC, MUAC^2^ | ✓ |  |  |  |  | ✓ |  | ✓ |  | ✓ |  |
| Lung function testing | ✓ |  |  |  |  | ✓ |  | ✓ |  | ✓ |  |
| T-cell subsets | ✓ |  |  |  |  | ✓ |  | ✓ |  | ✓ |  |
| Blood sample store | ✓ |  |  |  |  | ✓ |  | ✓ |  | ✓ |  |
| Psychosocial assessment^3^ | ✓ |  |  |  |  | ✓ |  | ✓ |  | ✓ |  |
| Pubertal Staging^4^ | ✓ |  |  |  |  |  |  | ✓ |  |  |  |
| Referral for TB investigations (if TB symptoms)^5^ | ✓ |  |  |  |  |  |  |  |  |  |  |

^1^Symptom checklist, hospitalisation, new diagnoses

^2^HC-head circumference; MUAC- mid-upper arm circumference;

^3^Knowledge of diagnosis, disclosure to others, schooling, guardianship, family HIV test

^4^For children aged 9 years or older

^5^Sputum (expectorated or induced), CXR, stool, urine will be collected as part of a collaborative study to be conducted by Martha Chipundiro (see Appendix 18.10)

**Table 7.2 Follow-up schedule for cohort participants who start ART**

|  |  | **Week** | | | | | | | | | | |
| --- | --- | --- | --- | --- | --- | --- | --- | --- | --- | --- | --- | --- |
|  | **0** | **2** | **4** | **6** | **8** | **12** | **24** | **36** | **48** | **60** | **72** |  |
| Start ART |  | ✓ |  |  |  |  |  |  |  |  |  |  |
| Nurse visit/collect medication^1^ |  | ✓ | ✓ |  | ✓ | ✓ | ✓ | ✓ | ✓ | ✓ | ✓ |  |
| Weight/Height | ✓ | ✓ | ✓ | ✓ | ✓ | ✓ | ✓ | ✓ | ✓ | ✓ | ✓ |  |
| HC, MUAC^2^ | ✓ |  |  |  |  |  | ✓ |  | ✓ |  | ✓ |  |
| Lung function testing | ✓ |  |  |  |  |  | ✓ |  | ✓ |  | ✓ |  |
| T-cell subsets | ✓ |  |  |  |  | ✓ | ✓ |  | ✓ |  | ✓ |  |
| HIV-1 viral load *(1 year post-ART)* |  |  |  |  |  |  |  |  | ✓ |  |  |  |
| Blood sample store^3^ | ✓ |  |  |  |  | ✓ |  |  | ✓ |  |  |  |
| Psychosocial assessment^4^ | ✓ |  |  |  |  |  | ✓ |  | ✓ |  | ✓ |  |
| Pubertal Staging^5^ | ✓ |  |  |  |  |  |  |  | ✓ |  |  |  |
| Adherence assessment |  |  |  |  |  | ✓ | ✓ |  | ✓ |  | ✓ |  |
| Referral for TB investigations (if TB symptoms)^6^ | ✓ |  |  |  |  |  |  |  |  |  |  |  |

^1^Clinical & toxicity symptom checklist, hospitalisation, new diagnoses

^2^HC-head circumference; MUAC- mid-upper arm circumference;

^3^Blood sample also taken at recruitment

^4^Knowledge of diagnosis, disclosure to others, schooling, guardianship, family HIV test

^5^For children aged 9 years or older

^6^Sputum (expectorated or induced), CXR, stool, urine will be collected as part of a collaborative study to be conducted by Martha Chipundiro (see Appendix 18.10)

## 7.5 Assessment of growth

All assessments of growth will be measured at baseline, before starting ART and at intervals specified in Table 7.1

Weight will be measured on every scheduled and unscheduled clinic visit using digital weight scales. Standing and sitting height (a measure of leg length)^72^ will be measured at every scheduled visit. Mid-upper arm circumference and head circumference will be measured six-monthly.

Puberty will be assessed annually using Tanner Staging.^73^ Tanner Staging will only be carried out in children who are ≥9 years. The following aspects of pubertal development will be recorded for males and females:

- Males- Testicular size (measured using a Prader orchidometer), Penile and pubic hair growth
- Females- Breast size and pubic hair growth; age of menarche

The Tanner pubertal stage recorded will be determined by breast development in females and testicular size in males

## 7.6 Assessment of lung function

Lung function will be carried out at baseline, before starting ART and on a six-monthly basis. Assessment will include:

- Spirometry
- Exercise tolerance -6-minute walk (only at baseline)
- Assessment of dyspnoea, using the MRC dyspnoea scale
- Pulse oximetry
- Respiratory rate

## Assessment of adherence

Adherence to ART will be assessed on every scheduled clinic visit, with a detailed assessment of average adherence made through a questionnaire administered to the participants/carer on a three monthly basis. Adherence will be assessed using self / caregiver report.

A brief standardized adherence measurement tool will be used to assess:

1. three day recall
2. pill taking patterns and
3. average monthly adherence estimate with a visual analogue scale

The adherence tool will be adapted from the WHO Generic Tools for Operational Research (WHO and Population Council). Average monthly (from visual analogue scale data) and mean annual adherence (from 3-day recall data) will be calculated. As an exploratory variable, adherence will be categorised using adherence categories such as <84%, 85-94%, and ≥95% representing low, medium, and high adherence.

In addition, pharmacy refill, dose and quantity of drugs dispensed by the clinic will also be documented.

## 7.8 Blood sampling

CD4 T-cell counts will be measured at baseline, at six monthly intervals, before starting ART and three months post-ART. HIV Viral load will be tested at one year post initiation of ART. Additional blood sampling (15mls) will be collected at baseline, at 6 monthly intervals among those who are not on ART. In those who start ART, blood will be taken at 3 months after starting ART and thereafter annually. Samples will be processed to obtain plasma, PBMC and DNA and stored for future immuno-genetic studies (Appendix 18.10).

a

# POST-PITC PREVALENCE SURVEY FOR UNDIAGNOSED HIV INFECTION

After two years of intervention, the prevalence of undiagnosed and diagnosed HIV infection in the 8 to 17 year age-group in the catchment area of intervention clinics will be measured through an HIV prevalence survey in a random selection of dwellings.

## 8.1 Sampling

A random sample of 90 Census Enumeration Areas (CEAs) located in the study suburbs where the study is taking place will be obtained. The list of CEAs will be obtained from the 2012 National Census (available through ZIMSTAT) with each CEA (defined as a as the smallest delimited census area) allocated a geo-code. Army camps, prisons, police, health institutions, educational institutions, hotels, guesthouses, lodges, refugee or squatter camps and other collective groups (such as nomadic, transit, and orphanages, old people’s homes) will be excluded from the sampling frame.

Simple random sampling will then be used to select the sample. The CEA codes will be used as a sampling frame. All households in the selected CEA will be enumerated. All the households in the CEA will be allocated an identification number. The number of households with children aged between 8 to 17 years will be determined. Any household within the selected CEA that has a child aged 8-17years as a household member will be eligible for participation in the prevalence survey. All the children in the selected household will be eligible for recruitment. Each child aged 8-17 years in the eligible household will be allocated a study number.

All households in the selected CEAs will be visited to confirm the exact location in the CEA, drop off information leaflet about the study and pre-book study interview time. The ZENITH survey team will visit the household for the actual interview at the pre-booked time. In the event that there are no children in the household or the household is vacant, the household will not be selected in the study. If the household is selected, the household head will be interviewed to obtain baseline economic and demographic information about the household and also obtain consent to interview the children. If consent is obtained and children are not at home or the appropriate guardian is not available on the day of visit, an appointment will be made and the household visited at the designated or appropriate time. A household will be visited a further two times if the interviewees are not present on the booked appointment time and date. Eligible children in the selected households will be visited twice if not present on the booked appointment. Where it is not possible to interview the household head or the child the reason for this will be recorded.

## 8.2 Participants

Eligibility criteria:

- Age ≥8 (day of their 8^th^ birthday or older) to 17 years (one day before their 18^th^ birthday). This age-group has been selected to ensure participants have had complete (2 year) exposure to the PITC intervention
- Written informed consent from the guardian
- Assent from the child

If there is more than one child in the household in the eligible age-range then all the children in the household will be tested. Inclusion of all children in the eligible age-group will provide an estimate of prevalence of HIV among siblings and allow exploration of “clustering” of HIV among children in households.

## 8.3 Collection of data and biological samples

The selected households will be visited and anonymised HIV testing will carried out on all children resident in the households, with guardian consent and participant assent.

An oral mucosal transudate (OMT) test will be collected from all participants to test for HIV antibodies. Participants will also be asked to provide a urine sample or dried blood spot (DBS) sample to test for antiretroviral drug concentration. This will identify those individuals who may under-report their HIV status.

Each household will be allocated an anonymous identification number and one form will be completed per household with provision for more than one child per household with separate HIV testing numbers identification numbers which are linked to the household per child. A questionnaire will be administered for each household whereby information on the following will be collected:-

- Demographics information on the household:
- The numbers and ages of all children of the household
- Socioeconomic variables, including difficulties in providing food, or in finding money to go to clinic if anyone in the household gets sick
- Demographic information on each child who participates:
- Age
- Sex
- Orphanhood status
- Grade in school (or not attending school and reason)
- Absenteeism from school due to illness
- History of HIV testing (including date of, place of and reason for test) and whether accessing HIV care/on ART

Risk factors for HIV infection at household level will be identified. If consent is declined or there is no guardian available in an eligible household to provide consent to participate, demographic information about the household will be requested from a key informant. This information will be used to define likely HIV status of children through imputation.

Undiagnosed and untreated HIV infection will be defined through self-reported past HIV-testing episodes, and self-reported chronic care medication (specifically, any ART, cotrimoxazole and IPT) being received at the time of interview. Clinical patient held records and any documentation of previous HIV test will be used to confirm the information given.

Guardians will be encouraged to bring children who have not tested before or cannot provide proof of testing to the PHCs for diagnostic testing and written information about HIV testing will be provided. Routine HIV testing for children and adolescents will be provided at the PHC of the suburb where the prevalence survey will be taking place supported by a ZENITH study research assistant and nurse. In addition, participants who test positive will automatically be offered HIV care and treatment at the same clinic. A CD4 count test will be done to ensure prompt assessment.

All participants of the prevalence survey will be offered free HIV testing (as described above). Each household will be randomised to receive 1) nothing, 2) a USD 2.00 incentive or 3) entry into a prize draw where they stand to get USD5.00, USD10.00 (this will be a draw so participants may get nothing) if the children in the selected households participate in the prevalence survey and come to the clinic for HIV counselling and testing. A flyer with benefits of HIV testing and the strategy the household has been randomised to will be given to participants. The clinic will carry out HIV testing according to National Guidelines with consent from the guardian (of participants aged <16 years) and assent from the participant (obtained verbally when they attend the clinic for HIV testing).

The standard operating procedures and forms used for the survey are listed in Appendix 18.2.

# OUTCOME EVALUATION

## 9.1 Definitions

*Missed appointment* Participant fails to attend a routine appointment within 7 days of a scheduled appointment or have not attended ART refill visit within 7 days of scheduled appointment

*Unscheduled visit* Any clinic visit that is not a scheduled appointment

*Pubertal delay* Appropriate Tanner Stage testicular size for boys and breast development for girls not achieved for age

*Stunting* Height-for-age z-scores using WHO reference Standards

*Wasting* Weight-for-age z-scores using WHO reference Standards

## 9.2 Capture of Study Endpoints

### 9.2.1 PITC

The following data on all children who attend the PHC aged between 6-16 years will be collected on a study form by the research assistant:

- Age and sex of child
- Age, sex of accompanying person
- PITC offered or not
- PITC accepted or refused and reason for refusal (i.e., either know status already or refusal of parental consent or inappropriate guardian)
- HIV-test result of child
- HIV test result of guardian

The data collection process will thus ensure that children eligible for PITC are not missed.

The following will be determined among children aged 6-15 years:

- number who attend the PHC
- number who attend without a guardian
- number who assent but guardians do not consent
- number who assent to testing with guardian consent
- number who test HIV-positive

### 9.2.2 Trial

The cumulative total number of children aged 6-15 years offered PITC who test HIV-positive and are recruited into the study under the trial arms will be determined.

The following data on the children who test HIV positive through PITC will be collected on study forms:

- Recruited or not recruited into the study
- Study arm recruited
- Routine clinic appointment dates on the clinic card and the actual visit date
- Any additional study visits
- On ART or not started
- Date started on ART
- CD4 count
- Viral Load post 12months ART
- On first vs second-line ART
- Pill count
- Adherence assessment result

Further, HIV-positive participants in each arm started on ART and date started will be used to determine if the participant has been on ART for a period of one year or more. The cumulative total number of participants who have been on ART for a period of ≥1 year will be determined.

A field will be generated which calculates the number of days between the routine appointment date and the actual routine visit date. If the difference is more than 7 days, an appointment will be classified as missed. The cumulative number of missed appointments for each participant will be calculated; the proportion of participants with more than two missed appointments will then be calculated for each arm.

Another ART outcome field will be generated which determines if the viral load is >400 copies/ml or if the participant has died within a period of one year on ART. If this is true, the ART outcome will be classified failure otherwise it will classified as success or unknown if the participant is lost to follow-up.

If the patient has not attended for an appointment for a period of >6 months, they will be followed up to ascertain the cause of this (death/move out of area/default /other). If alive and still in the area, cause of default will be determined as well as relevant clinical information. If agreeable the current viral load will be determined and the participant encouraged to re-attend for HIV care.

If the participant has died and the family is still in the area, the date of death will be recorded and a brief verbal autopsy conducted to ascertain the cause of death.

### 9.2.3 Post-PITC HIV Prevalence Survey

Participants’ guardians will be asked if they know their child(ren)’s HIV status and whether their child(ren) is/are on cotrimoxazole or ART. Data on whether a selected participant has accepted or refused anonymous HIV testing will be collected, as well as number of visits required before a guardian is identified. The following will be determined:

- Cumulative total of all participants found to be HIV-positive on anonymous HIV testing, who were known to be HIV-positive prior to the survey
- Cumulative total of all participants found to be HIV-positive on anonymous HIV testing, who were previously undiagnosed
- Cumulative total of all participants who were known to be HIV positive and are not currently on currently on cotrimoxazole or ART

### 9.2.4 Cohort Study

The following data will be recorded for participants enrolled in the cohort study:

- ART status
- CD4 count
- Weight, height, MUAC, HC, pubertal stage
- FEV1, FVC and FEV1: FVC
- Incident opportunistic infections, including TB
- Hospitalisations
- Deaths
- Side-effects of ART
- Adherence
- School attendance rates
- Disclosure
- Type or guardianship

## 9.3 Adverse Event Data Collection and Management

No serious adverse events (SAEs) are anticipated, but forms and a reporting protocol are in place, and any event deemed to be a serious adverse event will be reported to the Medical Research Council of Zimbabwe and the IRB of BRTI within 10 days.

The following incidents will also be systematically recorded and reported back to the respective IRBs: -

- breach of confidentiality following HIV diagnosis
- negative life-events following participation or HIV diagnosis
- withdrawal from participation in the study by patients
- needlestick injuries

# DATA COLLECTION AND MANAGEMENT

## 10.1 Data management

Data will be managed at the project offices in Biomedical Research and Training Institute. Data analysis will be carried out in-house in collaboration with LSHTM statisticians.

Data collection and entry will be as detailed in the SOPs (Appendix 18.8). All data will be entered into a Relational Access Database, using Optical Character Recognition (OCR) of paper forms, with validity checks before scanning, and a protocol for verification of scanned records.

Inconsistencies will be investigated and resolved. Trial and other data will be analysed in the interim and on completion of each respective stage of the study, with the aim being to have all data verified, double entered, checked and resolved according to the data analysis plan (Appendices). The final analyses will then use the combined study data.

Protocols for managing data without breach of confidentiality are in place. Access to the final data set will be limited to the Data Manager (Tsitsi Bandason), PI (Rashida Ferrand) and the LSHTM statistician (Helen Weiss). Sensitive information (including HIV results) will not be linked to personal identifiers in the final data set.

Data files will be kept in locked offices at BRTI during data entry, and in a locked data repository room for longer term storage.

## 10.2 Data capture forms and questionnaires

These are shown in the Appendices 18.2 to 18.7.

## 10.3 Data security

Hard copies of data and study documentation will be kept in locked offices, and long term storage will be in locked cupboards in a locked repository.

Electronic copies of data will be saved in password-protected files. NAS (Network attached Storage) Backup (http://www.nasbackup.com/index.php/Introduction) will be used to back data from PC onto NAS drives in a way that only the file differences are backed up every day. Additional permanent archives will be created by periodically backing up the data to two external hard drives every month. The hard drives will be stored in a locked filing cabinet away from the office by the PI.

## 10.4 Quality assurance

Data are checked for internal inconsistencies during verification and following data entry. Quality assurance protocols will be in place for each stage of the study, as detailed in individual SOPs.

# STATISTICAL CONSIDERATIONS

## 11.1 Assumptions re. survival of HIV-infected children

Data from prospective cohort studies in industrialised countries with follow-up beyond five years of age in the pre-ART era showed two patterns of disease progression in infants, rapid and slow progression. These data showed that approximately 20-30% of infected infants were rapid progressors and mortality approaches 100% by two years of age in this group. In the remainder, progression was much slower, with a substantial minority expected to survive childhood even without diagnosis and treatment.^74-81^ In Africa, there have been no natural history cohort studies assessing disease progression beyond five years of age.

A pooled cohort analysis estimated that the available data are most compatible with about one third being slow progressors whose median survival is at least 8 years with no upper limit to this estimate possible from the existing data.^9^ The remainder (fast-progressors) have high early mortality (median survival <2 years). Current estimates derive from survival models that combine the estimate of a third of vertically-infected infants being slow progressors with an assumption that median survival among this group will approximate that observed in children infected parenterally (i.e. approximate median survival of 16 years).^10, 12^ This equates to a 28% overall probability of HIV-infected infants surviving to 10 years, with approximately 1-3% of adolescents expected to be living with vertically-acquired HIV in Southern Africa.^12^

The main limitation of these models is that the median survival is extrapolated from that of children from developed countries infected through parenteral transmission, whose survival may well be quite different from those of vertically-infected slow-progressors in Africa. However, the projections are compatible with observed epidemiological data, suggesting that the model estimates provide a reasonably close approximation of the true natural history. In recent years, national population-based surveys in Southern Africa, however, have consistently reported high HIV prevalence rates of between 1-5% among older children.^12, 82-85^

For this project, it will not be possible to conduct an HIV prevalence survey prior to implementing PHC-based PITC, to investigate the burden of undiagnosed HIV. However, based on projections of HIV prevalence and the age distribution of children in HIV care in Zimbabwe, it is estimated that 80% of older children living with vertically-acquired HIV are currently undiagnosed.^8, 12, 14^

## 11.2 Sample size estimates

### 11.2.1 Individual-randomised trial

A sample of 175 children with newly diagnosed HIV per arm provides 80% power to detect a 45% reduction in proportion of children who miss ≥2 routine appointments in the follow up period in the intervention arm relative to the standard of care arm, given the most likely scenario of 30% missing in the control arm ^4, 86^, and allow 10% loss to follow-up, assuming a baseline HIV prevalence of 1.8%. The study also has 83% power to detect a 40% difference in proportions who miss ≥2 appointments, assuming that rates in the unexposed arm is 40%.

Studies have shown virological failure rates of 30-70% in older children. ^1, 87-88^ Assuming that at least 70% of the 175 children per arm with newly diagnosed HIV start ART, we expect 123 children per arm to be on ART. If the treatment failure/death rate among controls is 50%, the study will have 87% power to detect a 40% reduction in this rate. Given that our retention rate into the trial is better than expected, 350 participants will therefore provide adequate power to detect the difference in retention in care between the trial arms, even with the lower sample size.

### 11.2.2 Post-PITC Prevalence Survey Sample Size Estimates

We propose to survey children aged 8-17 years from approximately all households in 90 randomly selected CEAs to investigate the prevalence of undiagnosed HIV infection. Assuming an HIV prevalence of 3% among older children and a refusal rate of 25%, a sample size of 6,500 for the cross-sectional prevalence survey will include approximately 118 HIV infected individuals. We anticipate that the prevalence of undiagnosed HIV to drop by >50% as a result of optimal PITC implementation at PHCs. This should provide a 95% CI of around 40%-60% around a prevalence of undiagnosed HIV of 50% (i.e. precision of 10%),

### 11.2.3 Cohort Study

Participants registering for HIV care aged 6-15 years will be consecutively enrolled. We anticipate recruiting a minimum of 300 participants (out of the 350 participants who test HIV positive assuming up to 15% will be lost to follow-up). There would be 210 children on ART if 70% meet eligibility criteria to start ART.  The study will have about 80% power to detect a risk ratio of 1.4-2.2 if the proportion of ART-naïve children with the outcome of interest is at least 20% and 60-80% of the cohort takes ART (Table 11.1).

**Table 11.1: Power to detect risk ratio (RR) for outcomes in ART-naïve compared to**

**ART-exposed children for a given proportion of cohort on ART (total sample=300)**

| **Proportion on ART** | **No. on ART** | **Proportion with outcome in ART Naive** | **Proportion with outcome in ART exposed** | **RR** | **Power** |
| --- | --- | --- | --- | --- | --- |
| 60% | 180 | 50% | 35% | 1.4 | 80% |
| 60% | 180 | 20% | 9% | 2.2 | 82% |
| 70% | 210 | 50% | 35% | 1.4 | 80% |
| 70% | 210 | 30% | 16% | 1.8 | 86% |
| 70% | 210 | 20% | 9% | 2.2 | 86% |
| 80% | 240 | 50% | 36% | 1.4 | 86% |
| 80% | 240 | 20% | 9% | 2.2 | 90% |

# LABORATORY METHODS

## 12.1 Standard Operating Procedures

The Standard Operating Procedures used in the laboratory are shown in Appendix 18.7.

## 12.2 Diagnostic and anonymised HIV testing

For the HIV prevalence survey, participants will be asked to provide an oral mucosal test (OMT) sample for anonymised testing. The samples will be read on the same day. Each of the specimen containers will only bear the study ID of the participants. Diagnostic HIV testing in PHCs will be carried out according to National guidelines. Two rapid tests (Abbott Determine™ and SD Bioline™) will be run in parallel with a 3^rd^ rapid test (INSTI™) with used as a tiebreaker in case of discordant results between the first two tests.

Urine or DBS samples will be collected from participants to check for presence of ART. Urine samples of participants with HIV-positive OMT results will be individually tested for presence of ART. Urine samples of participants with HIV-negative result will be pooled (20 samples pooled) with individual testing of each sample should one pooled batch test ART-positive. DBS samples will be tested individually for ART.

## 12.3 CD4 count and viral load testing

CD4 count testing will be performed at the Beatrice Road Infectious Disease Hospital (BRIDH) laboratories using flow cytometry (PARTEC™). The BRIDH processes CD4 counts for all HIV-infected patients accessing care in City Health Services. Samples for Viral loads will be collected as DBS and outsourced for testing to the National Institute of Health Research (NIHR), Harare.

## 12.4 Processing and storage of blood specimens

Heparinised blood (10ml) will be collected at specified intervals during the study. Plasma, PBMC and DNA will be extracted and stored for use in immuno-genetic studies.

## 12.5 Safety issues

Staff training will be given to all employees given regarding universal precautions and avoidance of needle-stick injuries. A policy for needle-stick injuries is in place (Appendix 18.12). Residual specimens, used specimen containers and laboratory waste will be placed in sealed autoclaved bags, autoclaved, and incinerated at the end of each working day.

A quality management system is in place, with regular external audit of laboratory procedures.

# ADMINISTRATION AND MONITORING

## 13.1 Community Advisory Group

A Community Advisory Group (CAG) will be established in each study suburb to provide early feedback on any problems or community concerns, and enable rapid identification and rectification of any areas of concern. The Community Advisory Group will include members from the community and will meet initially as often as members feel appropriate, and then 6 monthly. Preliminary meetings with the CAGs will be conducted to introduce the study, and representatives of the CAGs will subsequently meet with the ZENITH Field Manager every three months.

## 13.2 Trial Steering Committee

A Trial Steering Committee (TSC) will be established and will advise on the conduct of the study and on logistical issues that arise during the project. The TSC will meet within three months of the start of recruitment and annually thereafter.

## 13.3 Good Clinical Practice

The principles of GCP will be adhered to, and the study team will have in-house GCP training. Modified GCP guidelines suitable for community-randomised public health interventions in resource poor settings will be developed for in-house training purposes.

## 13.4 Monitoring and audit

Project managers and supervisors will be responsible for day-to-day monitoring. Project meetings attended by all field staff will be held each fortnight, during which any problems encountered problems will be discussed and refresher training will be provided.

Internal audits will be conducted within three months of starting the intervention, and not less than annually during the intervention stage. These will include audit of study documentation, data storage, protocol adherence and SAE and incident reporting. A report will be circulated among the project team. Dr Ferrand will responsible for ensuring that problem areas identified during the audits are rectified, and for circulating details of actions to be taken by the study team.

The MRCZ carry out external audit of all research projects in Zimbabwe, and will conduct one or more audits of ZENITH during the tenure of the project. Otherwise, no external audit will be carried out. An external audit of BRTI finances is conducted annually and the ZENITH project will participate in this audit. In the context of the current trial, safety concerns are minimal; hence a Data Safety Monitoring Board (DSMB) has not been established and no interim analysis is planned.

# ETHICAL CONSIDERATIONS

## 14.1 Participant information leaflets

Leaflets will be produced at a level understandable by children and guardians. These will explain the nature of the trial, making it clear that the intervention is a research study. Separate leaflets will be produced for the anonymised HIV prevalence survey, providing information on where to access HIV testing, including names of health facilities and contact numbers of senior study personnel.

## 14.2 Consent

Written (or witnessed in the case of illiterate participants) informed consent /assent will be taken for: -

- Participation in formative research that will inform the design of the household support intervention (CBO, caregiver and HCW consent)
- Participation in the household support trial (child assent and guardian consent)
- Additional clinical assessment carried out as part of the clinical cohort study or assent (CBO, caregiver and HCW consent)
- The post-PITC HIV prevalence survey (CBO, caregiver and HCW consent)
- Monitoring and evaluation of the intervention through interview of VLWs (VLW consent)
- Storage of blood samples, including DNA (CBO, caregiver and HCW consent)

A guardian will be defined as someone who is over the age of 18 years who is responsible for the daily care, welfare and management of the rights of a minor (someone aged <16 years). The guardian does not necessarily have to be related to the child. If the biological parent is alive but absent (not providing daily care for the child), the individual responsible for the daily welfare of the child will be eligible to give consent. However, the guardian will be encouraged to contact the biological parent (if possible) for consent. If there is disagreement between the guardian and the child about participating in the study or undergoing HIV testing, both will be counselled until consensus is reached.

Children will require the consent of a guardian to undergo HIV testing. We request a waiver for written consent for provider-initiated HIV testing, as this is the recommended “standard of care” in Zimbabwe. An eligible child attending for primary care without a guardian will be given a flyer containing information about HIV testing services available at the clinic and encouraged to return for testing with their guardian. However, if the child has symptoms consistent with immunosuppression, they may be tested as part of clinical management with consent provided by a physician. Additionally, consent for testing could be applied for from social care services if it is in the child’s best interest to test and the child has no named guardian.

Emancipated minors i.e. participants who are married, have children or are the main care-givers for their younger siblings will be able to give consent independently for HIV testing and for participation in the study.

Those who test HIV-positive will be eligible for ongoing HIV care at the facility regardless of whether they consent to participate in the trial or in the cohort study.

## 14.3 Disclosure of HIV status

Post-test HIV counselling will be provided jointly to the guardian and to the participant. Children will be told their HIV status according to their level of understanding and maturity. The child’s HIV status will not disclosed to any health care worker without the consent of the guardian and child. A guardian attending with a child who has previously been tested but is unaware of his/her HIV status will be offered counselling and support to facilitate disclosure.

## 14.4 Ethics Committees providing review and approval

Application has been made to the ethics Committee of the London School of Hygiene And Tropical Medicine. Ethical approval has already been granted by the Institutional Review Board of the Biomedical Research and Training Institute and by the Medical Research Council of Zimbabwe (MRCZ).

## 14.5 Confidentiality

Participants’ identification data will be required for the registration process. Original databases will be stored with a field key file and a soft copy of the coded questionnaires. Alongside original data, anonymised databases will be created for use at the analysis stage.

## 14.6 Indemnity

The London School of Hygiene & Tropical Medicine holds Public Liability ("negligent harm") and Clinical Trial ("non-negligent harm") insurance policies which apply to this trial.

## 14.7 Trial registration

The trial has been registered with the Pan African Clinical Trials Registry (<http://www.pactr.org/>): Trial number-PACTR201212000442288

# STUDY TIME LINE

| **Activity (quarterly)** | **2012** | | | | **2013** | | | | | **2014** | | | | | **2015** | | | | | **2016** | | |
| --- | --- | --- | --- | --- | --- | --- | --- | --- | --- | --- | --- | --- | --- | --- | --- | --- | --- | --- | --- | --- | --- | --- |
|  | 2 | 3 | 4 | 1 | | 2 | 3 | 4 | 1 | | 2 | 3 | 4 | 1 | | 2 | 3 | 4 | 1 | | 2 | 3 |
| ***Miscellaneous*** |  |  |  |  | |  |  |  |  | |  |  |  |  | |  |  |  |  | |  |  |
| Equipment purchase and shipping |  | ✓ |  |  | |  |  |  |  | |  |  |  |  | |  |  |  |  | |  |  |
| Regulatory approvals | ✓ | ✓ |  |  | |  |  |  |  | |  |  |  |  | |  |  |  |  | |  |  |
| Staff recruitment and GCP training |  | ✓ | ✓ |  | |  |  |  |  | |  |  |  |  | |  |  |  |  | |  |  |
| Training in HIV management & Lung function |  |  | ✓ |  | |  |  |  |  | |  |  |  |  | |  |  |  |  | |  |  |
| ***PITC and VLW Trial*** |  |  |  |  | |  |  |  |  | |  |  |  |  | |  |  |  |  | |  |  |
| Develop data collection tools |  | ✓ | ✓ |  | |  |  |  |  | |  |  |  |  | |  |  |  |  | |  |  |
| Lead in period & attendance data from PHCs |  |  | ✓ |  | |  |  |  |  | |  |  |  |  | |  |  |  |  | |  |  |
| Pilot PITC in PHC |  |  | ✓ |  | |  |  |  |  | |  |  |  |  | |  |  |  |  | |  |  |
| PITC intervention |  |  |  | ✓ | | ✓ | ✓ | ✓ | ✓ | | ✓ | ✓ | ✓ |  | |  |  |  |  | |  |  |
| Cross-sectional HIV survey |  |  |  |  | |  |  |  |  | |  |  |  | ✓ | | ✓ |  |  |  | |  |  |
| Development of tools for formative research |  | ✓ |  |  | |  |  |  |  | |  |  |  |  | |  |  |  |  | |  |  |
| Fieldwork to inform VLW intervention |  | ✓ |  |  | |  |  |  |  | |  |  |  |  | |  |  |  |  | |  |  |
| Development of intervention |  |  | ✓ |  | |  |  |  |  | |  |  |  |  | |  |  |  |  | |  |  |
| Recruitment & training of VLW |  |  | ✓ |  | |  |  |  |  | |  |  |  |  | |  |  |  |  | |  |  |
| Enrolment into trial |  |  |  | ✓ | | ✓ | ✓ | ✓ | ✓ | |  |  |  |  | |  |  |  |  | |  |  |
| VLW-household intervention |  |  |  | ✓ | | ✓ | ✓ | ✓ | ✓ | | ✓ | ✓ | ✓ | ✓ | | ✓ | ✓ |  |  | |  |  |
| Process evaluation of intervention |  |  |  |  | | ✓ |  |  | ✓ | |  |  | ✓ |  | |  | ✓ |  |  | |  |  |
| Health economics |  |  |  |  | | ✓ | ✓ | ✓ | ✓ | | ✓ | ✓ | ✓ | ✓ | | ✓ | ✓ | ✓ |  | |  |  |
| Data analysis |  |  |  |  | |  | ✓ | ✓ | ✓ | | ✓ | ✓ | ✓ | ✓ | | ✓ | ✓ | ✓ |  | |  |  |
| Manuscript preparation & submission |  |  |  |  | |  |  |  |  | |  |  |  |  | |  | ✓ | ✓ | ✓ | | ✓ |  |
| ***Clinical Cohort Study*** |  |  |  |  | |  |  |  |  | |  |  |  |  | |  |  |  |  | |  |  |
| Cohort recruitment |  |  |  | ✓ | | ✓ | ✓ | ✓ | ✓ | |  |  |  |  | |  |  |  |  | |  |  |
| Cohort follow-up |  |  |  | ✓ | | ✓ | ✓ | ✓ | ✓ | | ✓ | ✓ | ✓ | ✓ | | ✓ | ✓ | ✓ |  | |  |  |
| Data analysis |  |  |  |  | |  |  |  |  | |  |  | ✓ | ✓ | | ✓ | ✓ | ✓ | ✓ | | ✓ | ✓ |
| Manuscript preparation & submission |  |  |  |  | |  |  |  |  | |  |  |  | ✓ | | ✓ |  |  |  | | ✓ | ✓ |

# DISSEMINATION

Results will be shared through six monthly meetings with the Department of City Health and the MOHCW. Results of interim data analysis will be presented at national research meetings, such as the annual research day held by the Institute of Continuing Health Education (ICHE) of the University of Zimbabwe and presented at international research meetings. Results will be prepared for publication in international peer reviewed scientific journals.

## 16.1 Policy for sharing data

Data will be primarily shared with scientific collaborators, but we will as far as possible facilitate data sharing with any group requesting access to individual patient records, using anonymised data. Priority will be given to local investigators and publicly-funded international researchers. Original databases will be stored with a field key file and a soft copy of the coded questionnaires. Composite databases will be stored with a field key file. Alongside original data, anonymised databases will be created and stored with all relevant data to facilitate any data transfer requests that are made subsequently.

Ethical clearance will be sought before data are transferred to other groups for secondary analysis. Data management and sharing policies will be in line with those of the MRCZ and the RCZ.

## 16.2 Strategy for communication with user communities

Results will be disseminated to communities through our Community Advisory Group, and will be presented at national and international meetings, and published in compliance with the Open Access policy of the Wellcome Trust.

# REFERENCES

1. Nachega JB, Hislop M, Nguyen H, Dowdy DW, Chaisson RE, Regensberg L, et al. Antiretroviral therapy adherence, virologic and immunologic outcomes in adolescents compared with adults in southern Africa. J Acquir Immune Defic Syndr. 2009 May 1;51(1):65-71.

2. Flynn PM, Rudy BJ, Douglas SD, Lathey J, Spector SA, Martinez J, et al. Virologic and immunologic outcomes after 24 weeks in HIV type 1-infected adolescents receiving highly active antiretroviral therapy. J Infect Dis. 2004 Jul 15;190(2):271-9.

3. Charles M, Noel F, Leger P, Severe P, Riviere C, Beauharnais CA, et al. Survival, plasma HIV-1 RNA concentrations and drug resistance in HIV-1-infected Haitian adolescents and young adults on antiretrovirals. Bull World Health Organ. 2008 Dec;86(12):970-7.

4. Horstmann E, Brown J, Islam F, Buck J, Agins BD. Retaining HIV-infected patients in care: Where are we? Where do we go from here? Clin Infect Dis. 2010 Mar 1;50(5):752-61.

5. AIDS Epidemic Update. Geneva, Switzerland: UNAIDS and WHO; 2009.

6. Newell ML, Coovadia H, Cortina-Borja M, Rollins N, Gaillard P, Dabis F. Mortality of infected and uninfected infants born to HIV-infected mothers in Africa: a pooled analysis. Lancet. 2004 Oct 2-8;364(9441):1236-43.

7. Ferrand RA, Bandason T, Musvaire P, Larke N, Nathoo K, Mujuru H, et al. Causes of acute hospitalization in adolescence: burden and spectrum of HIV-related morbidity in a country with an early-onset and severe HIV epidemic: a prospective survey. PLoS Med. 2010;7(2):e1000178.

8. Ferrand RA, Munaiwa L, Matsekete J, Bandason T, Nathoo K, Ndhlovu CE, et al. Undiagnosed HIV infection among adolescents seeking primary health care in Zimbabwe. Clin Infect Dis. 2010 Oct 1;51(7):844-51.

9. Marston M, Zaba B, Salomon JA, Brahmbhatt H, Bagenda D. Estimating the net effect of HIV on child mortality in African populations affected by generalized HIV epidemics. J Acquir Immune Defic Syndr. 2005 Feb 1;38(2):219-27.

10. Stover J, Walker N, Grassly NC, Marston M. Projecting the demographic impact of AIDS and the number of people in need of treatment: updates to the Spectrum projection package. Sex Transm Infect. 2006 Jun;82 Suppl 3:iii45-50.

11. Blanche S, Newell ML, Mayaux MJ, Dunn DT, Teglas JP, Rouzioux C, et al. Morbidity and mortality in European children vertically infected by HIV-1. The French Pediatric HIV Infection Study Group and European Collaborative Study. J Acquir Immune Defic Syndr Hum Retrovirol. 1997 Apr 15;14(5):442-50.

12. Ferrand RA, Corbett EL, Wood R, Hargrove J, Ndhlovu CE, Cowan FM, et al. AIDS among older children and adolescents in Southern Africa: projecting the time course and magnitude of the epidemic. Aids. 2009 Sep 24;23(15):2039-46.

13. Ferrand RA, Munaiwa L, Matsekete J, Bandason T, Nathoo K, Ndhlovu CE, et al. Undiagnosed HIV infection among Adolescents seeking Primary Health Care in Zimbabwe. Clinical Infectious Diseases. 2010;51:844-51.

14. Ferrand R, Lowe S, Whande B, Munaiwa L, Langhaug L, Cowan F, et al. Survey of children accessing HIV services in a high prevalence setting: time for adolescents to count? Bull World Health Organ. 2010 Jun;88(6):428-34.

15. Towards Universal Access: Scaling up priority HIV/AIDS interventions in the health sector: Progress Report 2010. Geneva: World Health Organization; 2010.

16. Stringer EM, Ekouevi DK, Coetzee D, Tih PM, Creek TL, Stinson K, et al. Coverage of nevirapine-based services to prevent mother-to-child HIV transmission in 4 African countries. Jama. 2010 Jul 21;304(3):293-302.

17. Ferrand RA, Desai SR, Hopkins C, Elston CM, Copley SJ, Nathoo K, et al. Chronic Lung Disease in Adolescents With Delayed Diagnosis of Vertically Acquired HIV Infection. Clin Infect Dis. 2012 Jul;55(1):145-52.

18. Rylance J, Mwalukomo T, Rylance S, Matchere P, Tindwa D, Webb EL, et al. Lung Function and Bronchodilator Response in Perinatally HIV-Infected Malawian Adolescents. 19th Conference on Retroviruses and Opportunistic Infections. Seattle, USA; 2012.

19. Kastelijn EA, van Moorsel CH, Ruven HJ, Karthaus V, Kwakkel-van Erp JM, van de Graaf EA, et al. Genetic polymorphisms in MMP7 and reduced serum levels associate with the development of bronchiolitis obliterans syndrome after lung transplantation. J Heart Lung Transplant. 2010 Jun;29(6):680-6.

20. Becroft DM. Bronchiolitis obliterans, bronchiectasis, and other sequelae of adenovirus type 21 infection in young children. J Clin Pathol. 1971 Feb;24(1):72-82.

21. Tiddens H, Silverman M, Bush A. The role of inflammation in airway disease: remodeling. Am J Respir Crit Care Med. 2000 Aug;162(2 Pt 2):S7-S10.

22. Lazarus EM, Otwombe K, Mohapi L, Cescon A, Violari A, Laher F. Effect of baseline immunological condition, virological response and duration of HAART on growth in HIV-infected adolescents. Eighteenth International AIDS Conference. Vienna; July 2010.

23. Bakeera-Kitaka S, McKellar M, Snider C, Kekitiinwa A, Piloya T, Musoke P, et al. Antiretroviral therapy for HIV-1 infected adolescents in Uganda: Assessing the impact on growth and sexual maturation Journal of Pediatric Infectious Diseases. 2008;3(2):97-104.

24. Brenchley JM, Price DA, Douek DC. HIV disease: fallout from a mucosal catastrophe? Nat Immunol. 2006 Mar;7(3):235-9.

25. Connolly NC, Riddler SA, Rinaldo CR. Proinflammatory cytokines in HIV disease-a review and rationale for new therapeutic approaches. AIDS Rev. 2005 Jul-Sep;7(3):168-80.

26. Neuhaus J, Jacobs Jr DR, Baker JV, Calmy A, Duprez D, La Rosa A, et al. Markers of Inflammation, Coagulation, and Renal Function Are Elevated in Adults with HIV Infection. J Infect Dis. 2010 May 6.

27. Foster G, Shakespeare R, Chinemana F, Jackson H, Gregson S, Marange C, et al. Orphan prevalence and extended family care in a peri-urban community in Zimbabwe AIDS Care. 1995;7(1):3-17.

28. Antiretroviral therapy for HIV infection in infants and children: Towards universal access. Recommendations for a public health approach: 2010 revision. Geneva, Switzerland: World Health Organization; 2010.

29. Kekitiinwa A, Lee KJ, Walker AS, Maganda A, Doerholt K, Kitaka SB, et al. Differences in factors associated with initial growth, CD4, and viral load responses to ART in HIV-infected children in Kampala, Uganda, and the United Kingdom/Ireland. J Acquir Immune Defic Syndr. 2008 Dec 1;49(4):384-92.

30. Tayler-Smith K, Zachariah R, Massaquoi M, Manzi M, Pasulani O, van den Akker T, et al. Unacceptable attrition among WHO stages 1 and 2 patients in a hospital-based setting in rural Malawi: can we retain such patients within the general health system? Trans R Soc Trop Med Hyg. 2010 May;104(5):313-9.

31. Braitstein P, Brinkhof MW, Dabis F, Schechter M, Boulle A, Miotti P, et al. Mortality of HIV-1-infected patients in the first year of antiretroviral therapy: comparison between low-income and high-income countries. Lancet. 2006 Mar 11;367(9513):817-24.

32. Murphy DA, Belzer M, Durako SJ, Sarr M, Wilson CM, Muenz LR. Longitudinal antiretroviral adherence among adolescents infected with human immunodeficiency virus. Arch Pediatr Adolesc Med. 2005 Aug;159(8):764-70.

33. Flynn PM, Rudy BJ, Lindsey JC, Douglas SD, Lathey J, Spector SA, et al. Long-term observation of adolescents initiating HAART therapy: three-year follow-up. AIDS Res Hum Retroviruses. 2007 Oct;23(10):1208-14.

34. Nyamukapa CA, Gregson S, Lopman B, Saito S, Watts HJ, Monasch R, et al. HIV-associated orphanhood and children's psychosocial distress: theoretical framework tested with data from Zimbabwe. Am J Public Health. 2008 Jan;98(1):133-41.

35. Gregson S, Nyamukapa CA, Garnett GP, Wambe M, Lewis JJ, Mason PR, et al. HIV infection and reproductive health in teenage women orphaned and made vulnerable by AIDS in Zimbabwe. AIDS Care. 2005;17(7):785-94.

36. Birungi H, Obare F, Mugisha JF, Evelia H, Nyombi J. Preventive service needs of young people perinatally infected with HIV in Uganda. AIDS Care. 2009 Jun;21(6):725-31.

37. Cameron F. Teenagers with diabetes--management challenges. Aust Fam Physician. 2006 Jun;35(6):386-90.

38. Roca B. Adverse drug reactions to antiretroviral medication. Front Biosci. 2009;14:1785-92.

39. Kemp JR, Mann G, Simwaka BN, Salaniponi FM, Squire SB. Can Malawi's poor afford free tuberculosis services? Patient and household costs associated with a tuberculosis diagnosis in Lilongwe. Bull World Health Organ. 2007 Aug;85(8):580-5.

40. Birdthistle IJ, Floyd S, Machingura A, Mudziwapasi N, Gregson S, Glynn JR. From affected to infected? Orphanhood and HIV risk among female adolescents in urban Zimbabwe. Aids. 2008 Mar 30;22(6):759-66.

41. Nyamukapa C, Gregson S. Extended family's and women's roles in safeguarding orphans' education in AIDS-afflicted rural Zimbabwe. SocSciMed. 2005;60(10):2155-67.

42. Zachariah R, Teck R, Buhendwa L, Fitzerland M, Labana S, Chinji C, et al. Community support is associated with better antiretroviral treatment outcomes in a resource-limited rural district in Malawi. Trans R Soc Trop Med Hyg. 2007 Jan;101(1):79-84.

43. Yeap AD, Hamilton R, Charalambous S, Dwadwa T, Churchyard GJ, Geissler PW, et al. Factors influencing uptake of HIV care and treatment among children in South Africa - a qualitative study of caregivers and clinic staff. AIDS Care. 2010 Sep;22(9):1101-7.

44. Obermeyer CM, Osborn M. The utilization of testing and counseling for HIV: a review of the social and behavioral evidence. Am J Public Health. 2007 Oct;97(10):1762-74.

45. Mills EJ, Nachega JB, Bangsberg DR, Singh S, Rachlis B, Wu P, et al. Adherence to HAART: a systematic review of developed and developing nation patient-reported barriers and facilitators. PLoS Med. 2006 Nov;3(11):e438.

46. Vreeman RC, Nyandiko WM, Ayaya SO, Walumbe EG, Marrero DG, Inui TS. The perceived impact of disclosure of pediatric HIV status on pediatric antiretroviral therapy adherence, child well-being, and social relationships in a resource-limited setting. AIDS Patient Care STDS. 2010 Oct;24(10):639-49.

47. Dahab M, Charalambous S, Hamilton R, Fielding K, Kielmann K, Churchyard GJ, et al. "That is why I stopped the ART": patients' & providers' perspectives on barriers to and enablers of HIV treatment adherence in a South African workplace programme. BMC Public Health. 2008;8:63.

48. Veinot TC, Flicker SE, Skinner HA, McClelland A, Saulnier P, Read SE, et al. "Supposed to make you better but it doesn't really": HIV-positive youths' perceptions of HIV treatment. Journal of Adolescent Health. 2006;38(3):261-7.

49. Lawn JE, Rohde J, Rifkin S, Were M, Paul VK, Chopra M. Alma-Ata 30 years on: revolutionary, relevant, and time to revitalise. Lancet. 2008 Sep 13;372(9642):917-27.

50. Lewin S, Munabi-Babigumira S, Glenton C, Daniels K, Bosch-Capblanch X, van Wyk BE, et al. Lay health workers in primary and community health care for maternal and child health and the management of infectious diseases. Cochrane Database Syst Rev. 2010(3):CD004015.

51. Schneider H, Lehmann U. Lay health workers and HIV programmes: implications for health systems. AIDS Care. 2010;22 Suppl 1:60-7.

52. Hermann K, Van Damme W, Pariyo GW, Schouten E, Assefa Y, Cirera A, et al. Community health workers for ART in sub-Saharan Africa: learning from experience--capitalizing on new opportunities. Hum Resour Health. 2009;7:31.

53. Nsigaye R, Wringe A, Roura M, Kalluvya S, Urassa M, Busza J, et al. From HIV diagnosis to treatment: evaluation of a referral system to promote and monitor access to antiretroviral therapy in rural Tanzania. J Int AIDS Soc. 2009;12(1):31.

54. Kabore I, Bloem J, Etheredge G, Obiero W, Wanless S, Doykos P, et al. The Effect of Community-Based Support Services on Clinical Efficacy and Health-Related Quality of Life in HIV/AIDS Patients in Resource-Limited Settings in Sub-Saharan Africa. AIDS Patient Care and STDs. 2010;24(9):581-94.

55. Campbell C, Skovdal M, Mupambireyi Z, Madanhire C, Nyamukapa C, Gregson S. Building adherence-competent communities: Factors promoting children's adherence to anti-retroviral HIV/AIDS treatment in rural Zimbabwe. Health & Place. 2012;18(2):123-31.

56. Vreeman RC, Nyandiko WM, Ayaya SO, Walumbe EG, Marrero DG, Inui TS. Factors Sustaining Pediatric Adherence to Antiretroviral Therapy in Western Kenya. Qual Health Res. 2009 December 1, 2009;19(12):1716-29.

57. Wouters E, Van Damme W, Van Loon F, van Rensburg D, Meulemans H. Public-sector ART in the Free State Province, South Africa: Community support as an important determinant of outcome. Social Science & Medicine. 2009;69(8):1177-85.

58. Merten S, Kenter E, McKenzie O, Musheke M, Ntalasha H, Martin-Hilber A. Patient-reported barriers and drivers of adherence to antiretrovirals in sub-Saharan Africa: a meta-ethnography. Trop Med Int Health. 2010 Jun;15 Suppl 1:16-33.

59. Ware NC, Idoko J, Kaaya S, Biraro IA, Wyatt MA, Agbaji O, et al. Explaining Adherence Success in Sub-Saharan Africa: An Ethnographic Study. PLoS Med. 2009;6(1):e1000011.

60. Fatti G, Meintjes G, Shea J, Eley B, Grimwood A. Improved Survival and Antiretroviral Treatment Outcomes in Adults Receiving Community-Based Adherence Support: Five-Year Results from a Multicentre Cohort Study in South Africa. JAIDS Journal of Acquired Immune Deficiency Syndromes. e-pub;Publish Ahead of Print:10.1097/QAI.0b013e31826a6aee.

61. O’Laughlin K, Wyatt M, Kaaya S, Bangsberg D, Ware N. How Treatment Partners Help: Social Analysis of an African Adherence Support Intervention. AIDS and Behavior. 2012;16(5):1308-15.

62. Busza J, Walker D, Hairston A, Gable A, Pitter C, Lee S, et al. Community-based approaches for prevention of mother to child transmission in resource-poor settings: a social ecological review. J Int AIDS Soc. 2012;15(4):1-11.

63. Etienne M, Burrows L, Osotimehin B, Macharia T, Hossain B, Redfield RR, et al. Situational analysis of varying models of adherence support and loss to follow up rates; findings from 27 treatment facilities in eight resource limited countries. Tropical Medicine & International Health. 2010;15:76-81.

64. Harries AD, Zachariah R, Lawn SD, Rosen S. Strategies to improve patient retention on antiretroviral therapy in sub-Saharan Africa. Tropical Medicine & International Health. 2010;15:70-5.

65. Hermann K, Van Damme W, Pariyo G, Schouten E, Assefa Y, Cirera A, et al. Community health workers for ART in sub-Saharan Africa: learning from experience - capitalizing on new opportunities. Human Resources for Health. 2009;7(1):31.

66. Decroo T, Van Damme W, Kegels G, Remartinez D, Rasschaert F. Are Expert Patients an Untapped Resource for ART Provision in Sub-Saharan Africa? AIDS Res Treat. 2012;2012:749718.

67. Gusdal AK, Obua C, Andualem T, Wahlström R, Chalker J, Fochsen G, et al. Peer counselors' role in supporting patients' adherence to ART in Ethiopia and Uganda. AIDS Care. 2011 2011/06/01;23(6):657-62.

68. Van Winghem J, Telfer B, Reid T, Ouko J, Mutunga A, Jama Z, et al. Implementation of a comprehensive program including psycho-social and treatment literacy activities to improve adherence to HIV care and treatment for a pediatric population in Kenya. BMC Pediatr. 2008;8:52.

69. Skovdal M, Campbell C, Madanhire C, Nyamukapa C, Gregson S. Challenges faced by elderly guardians in sustaining the adherence to antiretroviral therapy in HIV-infected children in Zimbabwe. AIDS Care. 2011 2011/08/01;23(8):957-64.

70. Grimwood A, Fatti G, Mothibi E, Malahlela M, Shea J, Eley B. Community adherence support improves programme retention in children on antiretroviral treatment: a multicentre cohort study in South Africa. J Int AIDS Soc. 2012;15(2):1-9.

71. Schneider H, Hlophe H, van Rensburg D. Community health workers and the response to HIV/AIDS in South Africa: tensions and prospects. Health Policy and Planning. 2008 May 1, 2008;23(3):179-87.

72. Cole TJ. The secular trend in human physical growth: a biological view. Econ Hum Biol. 2003 Jun;1(2):161-8.

73. Tindyebwa D, Kayita J, Musoke P, Eley B, Nduati R, H C. Handbook on Paediatric AIDS in Africa. Kampala, Uganda: African Network for the Care of Children; 2004.

74. Tovo PA, de Martino M, Gabiano C, Cappello N, D'Elia R, Loy A, et al. Prognostic factors and survival in children with perinatal HIV-1 infection. The Italian Register for HIV Infections in Children. Lancet. 1992 May 23;339(8804):1249-53.

75. Blanche S, Tardieu M, Duliege A, Rouzioux C, Le Deist F, Fukunaga K, et al. Longitudinal study of 94 symptomatic infants with perinatally acquired human immunodeficiency virus infection. Evidence for a bimodal expression of clinical and biological symptoms. Am J Dis Child. 1990 Nov;144(11):1210-5.

76. Mayaux MJ, Burgard M, Teglas JP, Cottalorda J, Krivine A, Simon F, et al. Neonatal characteristics in rapidly progressive perinatally acquired HIV-1 disease. The French Pediatric HIV Infection Study Group. Jama. 1996 Feb 28;275(8):606-10.

77. Scott GB, Hutto C, Makuch RW, Mastrucci MT, O'Connor T, Mitchell CD, et al. Survival in children with perinatally acquired human immunodeficiency virus type 1 infection. N Engl J Med. 1989 Dec 28;321(26):1791-6.

78. Auger I, Thomas P, De Gruttola V, Morse D, Moore D, Williams R, et al. Incubation periods for paediatric AIDS patients. Nature. 1988 Dec 8;336(6199):575-7.

79. Commenges D, Alioum A, Lepage P, Van de Perre P, Msellati P, Dabis F. Estimating the incubation period of paediatric AIDS in Rwanda. Aids. 1992 Dec;6(12):1515-20.

80. Stiehm ER. Newborn factors in maternal-infant transmission of pediatric HIV infection. J Nutr. 1996 Oct;126(10 Suppl):2632S-6S.

81. Grubman S, Gross E, Lerner-Weiss N, Hernandez M, McSherry GD, Hoyt LG, et al. Older children and adolescents living with perinatally acquired human immunodeficiency virus infection. Pediatrics. 1995 May;95(5):657-63.

82. Bandason T, Langhaug L, Makamba M, Laver S, Hatzhold K, Mahere S, et al. Burden of HIV and Feasibility of School-Linked HIV Testing Among Primary School Children in Harare, Zimbabwe. 18th Conference of Retroviruses and Opportunistic Infections Boston, USA; 2011.

83. Shisana O, Rehle T, Simbayi L, Zuma K, Jooste S, Pillay-van-Wyk V, et al. South African national HIV prevalence, incidence, behaviour and communication survey 2008: a turning tide among teenagers? Cape Town: HSRC Press; 2009.

84. Swaziland Demographic and Health Survey, 2006-2007. Final report. Swaziland: Central Statistics Office and Measure DHS; 2008 June 2007.

85. Gomo E, Rusakaniko S, Mashange W, Mutswanga J, Chandiwana B, Munyati S. Household survey of HIV-prevalence and behaviour in Chimanimani District, Zimbabwe Cape Town, South Africa: Human Social Research Council; 2005.

86. Mugavero MJ, Lin HY, Willig JH, Westfall AO, Ulett KB, Routman JS, et al. Missed visits and mortality among patients establishing initial outpatient HIV treatment. Clin Infect Dis. 2009 Jan 15;48(2):248-56.

87. Amornkul PN, Vandenhoudt H, Nasokho P, Odhiambo F, Mwaengo D, Hightower A, et al. HIV prevalence and associated risk factors among individuals aged 13-34 years in Rural Western Kenya. PLoS ONE. 2009;4(7):e6470.

88. Flynn PM, Rudy BJ, Douglas SD, Lathey J, Spector SA, Martinez J, et al. Virologic and immunologic outcomes after 24 weeks in HIV type 1-infected adolescents receiving highly active antiretroviral therapy. Journal of Infectious Diseases. 2004;190(2):271-9.

# Appendices

Kept as separate folders in the electronic copy of the protocol

## 18.1 Map of Study sites

## 18.2 PITC and HIV survey SOPs

## 18.3 Formative Study Protocol and SOPs

## 18.4 Trial SOPs

## 18.5 VLW Intervention

## 18.6 Cohort Study SOPs

## 18.7 Laboratory SOPs

## 18.8 Data handling and management

## 18.9 Community Advisory Groups

## 18.10 Other studies arising from the project

## 18.11 Ethical approval and consent forms

## 18.12 Needlestick injury policy

## 18.13 Zimbabwe HIV testing and treatment Guidelines

## 18.14 Study Forms

1. DETECTB: a cluster-randomised trial Survey conducted in the high-density Western residential suburbs of Harare of two active case-finding strategies for tuberculosis [↑](#footnote-ref-2)
