# Supplementary material for: Community burden of undiagnosed HIV infection among adolescents in Zimbabwe following primary healthcare-based provider-initiated HIV testing and counselling: A cross-sectional survey
Source: PLoS Med. 2017 Jul 25;14(7):e1002360. doi: 10.1371/journal.pmed.1002360 (PMC5526522; doi:10.1371/journal.pmed.1002360)
Supplement: S6 Text — (DOCX) [file pmed.1002360.s010.docx]

**
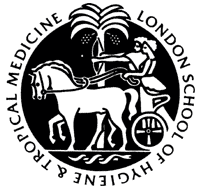
**

**ZENITH**

**Zimbabwe study for Enhancing Testing and Improving Treatment of HIV in Children**

**Prevalence Survey**

**Statistical Analysis Plan V1.2**

**7^th^ April 2016**

**Funder Wellcome Trust, UK**

**Principal Investigator Dr Rashida A Ferrand**

**Host Institute Biomedical Research and Training Institute, Zimbabwe**

**TABLE OF CONTENTS**

[1 OBJECTIVES AND STUDY DESIGN OVERVIEW 3](#_Toc447807352)

[1.1 Aims and Objectives 3](#_Toc447807353)

[1.2 Study Design Overview 3](#_Toc447807354)

[2 Cross-sectional prevalence survey 4](#_Toc447807355)

[2.1 Inclusion and exclusion criteria 4](#_Toc447807356)

[2.2 Sampling 4](#_Toc447807357)

[2.3 Sample size estimates 4](#_Toc447807358)

[2.4 Data collection 4](#_Toc447807359)

[2.5 Outcome measures 5](#_Toc447807360)

[2.5.1 Primary outcome measure 5](#_Toc447807361)

[2.5.2 Secondary outcome measure 5](#_Toc447807362)

[2.6 Statistical analyses 6](#_Toc447807363)

[3 Figure 1: Flow of participant recruitment 7](#_Toc447807364)

# OBJECTIVES AND STUDY DESIGN OVERVIEW

## Aims and Objectives

The broad aim of this study to investigate whether delivery of provider-initiated HIV testing and counselling (PITC) at primary care clinics reduces the burden of undiagnosed HIV infection at community level in older children and adolescents.

The specific objectives are:

1. To investigate the prevalence of undiagnosed HIV infection in the catchment areas of seven primary care clinics that offer PITC
2. To estimate the prevalence of HIV infection by age-group
3. To investigate the sensitivity of the oral mucosal HIV test (OMT) in detecting HIV infection
4. To investigate the accuracy of self-report of previous HIV testing
5. To investigate factors associated with undiagnosed HIV infection

## Study Design Overview

The study will be conducted in the main PHCs of seven suburbs in Southwest Harare. Each participating PHC will offer PITC routinely to 6-15 years olds over a two year period. Children who test HIV-positive through PITC will be offered HIV care at the same PHC. At the end of two years of implementation of PITC, an anonymised HIV prevalence survey of 8-17 years olds from randomly selected dwellings in the suburbs served by the study PHCs will be conducted. The prevalence of *undiagnosed* HIV infection in the community following two years of PITC will provide an indication of the effectiveness of primary-care based routine HIV testing in providing high enough coverage of HIV diagnosis in this age-group (>80% of children diagnosed). The survey will also provide data on overall HIV prevalence by age in the considered age-group.

The uptake of HIV testing, and prevalence and yield of HIV infection in the seven study PHCs and the barriers to uptake have already been reported. This document focuses on the analysis of the cross-sectional survey following 2 years of PITC delivered at the study clinics.

# Cross-sectional prevalence survey

## Inclusion and exclusion criteria

The inclusion criteria for participation in the survey:

- Member of a household (HH) located in the randomly selected census enumeration area (CEA)
- Age ≥8 (day of their 8^th^ birthday or older) to 17 years (one day before their 18^th^ birthday). This age-group has been selected to ensure participants have had complete (2 year) exposure to the PITC intervention
- Written informed consent from the guardian
- Assent from the child

The exclusion criteria for participation in the trial will be:

- Not likely to be available in the HH within the next 2 weeks
- Not found in the HH after 2 home visits

## Sampling

A random sample of 130 Census Enumeration Areas (CEAs) in the seven study suburbs where will be obtained. The list of CEAs will be obtained from the 2012 National Census (available through ZIMSTAT) with each CEA (defined as a as the smallest delimited census area) allocated a geo-code. The CEA codes will be used as a sampling frame and simple random sampling will then be used to select the sample. All households in the selected CEA will be enumerated and households with a member aged 8-17 year olds will be eligible to participate and each child aged 8-17 years in the eligible household will be eligible to participate. This provides a self-weighting sample.

## Sample size estimates

We propose to survey children aged 8-17 years from approximately all households in the randomly selected CEAs. Assuming an HIV prevalence of 3% among older children and a refusal rate of 25%, a sample size of 6,500 for the cross-sectional prevalence survey will include approximately 118 HIV infected individuals. We anticipate that the prevalence of undiagnosed HIV to drop by >50% as a result of optimal PITC implementation at PHCs. This should provide a 95% CI of around 40%-60% around a prevalence of undiagnosed HIV of 50% (i.e. precision of 10%),

## Data collection

- Household level data (collected at enumeration stage)

No of HH members including those aged <8, 8-17 yrs, >17 years

Age and educational level of HH head

HH income

Deaths in the HH in past 12 months

- Individual level data collection (only in those enrolled into survey)

Age group (8-12 and 13-17 years)

Sex

Orphanhood

Type of caregiver

Marital status and current schooling

Length of residence in current HH

Clinical variables (past TB, past hospitalisation, self-report of poor health, skin problems)

Whether previous HIV testing

Date of last HIV test

Location of past HIV test

Proof of HIV test (result)

Taking ART or cotrimoxazole

Oral Mucosal HIV Test (OMT) result

Urine or DBS ART (UDA): whether ART found in urine or DBS

Urine or DBS ART results are only available in 50% of the sample.

## Outcome measures

### Primary outcome measure

- Among those with a UDA sample: Proportion of undiagnosed HIV infection among those HIV positive (overall and by age group)

**Table 1: Definition of undiagnosed HIV and previously known HIV positive status**

|  | **Negative LAM result** | | **Positive LAM result** | | **LAM not done** | |
| --- | --- | --- | --- | --- | --- | --- |
| **HIV self-report** | **OMT+** | **OMT-** | **OMT+** | **OMT-** | **OMT+** | **OMT-** |
| **Positive** | Known | Known | Known | Known | Known | Known |
| **Negative** | Undiagnosed | Negative | Known | Known | Undiagnosed | Negative |
| **Don’t know** | Undiagnosed | Negative | Known | Known | Undiagnosed | Negative |

### Secondary outcome measure

- Proportion of undiagnosed HIV in participants with OMT only, overall and by age group
- Proportion of participants who under-reported positive HIV status (self-report negative/LAM positive)
- Sensitivity of OMT test (OMT positive/LAM positive)
- Factors associated with undiagnosed HIV infection (in those with UDA)
- Factors associated with HIV infection (in all participants)
- Comparison of characteristics of participants with and without UDA

## Statistical analyses

Prevalence and 95%CI of undiagnosed HIV will be estimated allowing for clustering with suburb as a strata and CEA as a primary sampling unit.

Factors associated with undiagnosed HIV infection (in those with UDA) and HIV infection (in all participants) will be analysed using mixed effects logistic regression, allowing for CEA and household as random effects.
